# Identification of a Transcription Factor That Regulates Host Cell Exit and Virulence of *Mycobacterium tuberculosis*

**DOI:** 10.1371/journal.ppat.1005652

**Published:** 2016-05-18

**Authors:** Lalitha Srinivasan, Serdar A. Gurses, Benjamin E. Hurley, Jessica L. Miller, Petros C. Karakousis, Volker Briken

**Affiliations:** 1 Department of Cell Biology and Molecular Genetics, University of Maryland, College Park, Maryland, United States of America; 2 Department of Medicine, Johns Hopkins University School of Medicine, Baltimore, Maryland, United States of America; University of Massachusetts Medical School, UNITED STATES

## Abstract

The interaction of *Mycobacterium tuberculosis* (Mtb) with host cell death signaling pathways is characterized by an initial anti-apoptotic phase followed by a pro-necrotic phase to allow for host cell exit of the bacteria. The bacterial modulators regulating necrosis induction are poorly understood. Here we describe the identification of a transcriptional repressor, Rv3167c responsible for regulating the escape of Mtb from the phagosome. Increased cytosolic localization of MtbΔ*Rv3167c* was accompanied by elevated levels of mitochondrial reactive oxygen species and reduced activation of the protein kinase Akt, and these events were critical for the induction of host cell necrosis and macroautophagy. The increase in necrosis led to an increase in bacterial virulence as reflected in higher bacterial burden and reduced survival of mice infected with MtbΔ*Rv3167c*. The regulon of Rv3167c thus contains the bacterial mediators involved in escape from the phagosome and host cell necrosis induction, both of which are crucial steps in the intracellular lifecycle and virulence of Mtb.

## Author Summary

*Mycobacterium tuberculosis* (Mtb), the causative agent of tuberculosis, is a highly successful human pathogen. Following entry into host phagocytic cells, Mtb resides within a modified phagosomal compartment and inhibits apoptotic host cell death. Recent studies have demonstrated that Mtb eventually translocates from the phagosomal compartment to the cytosol. This event is followed by the induction of necrotic host cell death allowing the bacteria to exit the host cell and infect naive cell populations. Our study adds to this relatively unexplored aspect of Mtb pathogenesis by revealing that the transcriptional repressor *Rv3167c* of Mtb negatively regulates phagosomal escape and host cell necrosis. We furthermore demonstrate that the increased necrosis induction by the Mtb mutant strain deficient in *Rv3167c* required elevated reactive oxygen species levels within host cell mitochondria and reduced activation of the protein kinase Akt. In addition, the increased virulence of the Mtb mutant strain observed after aerosol infection of mice strengthens the link between the ability of the bacteria to induce host cell necrosis and virulence. The Mtb genes negatively regulated by Rv3167c are thus potential virulence factors that can be targeted for drug and vaccine development.

## Introduction

Apoptosis is a major programmed cell death pathway but now it is well established that necrosis can also be induced via defined signal transduction pathways [1,2]. The importance of apoptosis in host defense against pathogens is well described [3,4]. In contrast, the function of programmed necrosis in host resistance or susceptibility to pathogens is still an open question in many cases and may depend upon the context of the infection and the pathogen [5]. For instance, the RIPK1/3 necrosis pathway acts as a back-up mechanism of death induction in cells infected with viruses that are able to inhibit host cell apoptosis [6]. Consequently, programmed necrosis is associated with increased host resistance against viral pathogens in the case of vaccinia virus, adenovirus and MCMV [5,6]. Nevertheless, for the influenza A virus, programmed necrosis leads to increased pathology and host susceptibility [7]. Limited results are available for interaction of bacterial pathogens with host cell necrosis pathways but similar to viral pathogens the role of programmed necrosis may vary depending upon the pathogen. Enteropathogenic *Escherichia coli* can inhibit RIPK3-dependent necrosis via the glycosyl transferase NleB and this activity is important for bacterial virulence [8,9]. In contrast, IRF-3-dependent necrosis induction by *Listeria monocytogenes* promotes pathogen dissemination and virulence [10].

The interaction of wild-type *Mycobacterium tuberculosis* (Mtb) with its host cell in regard to cell death signaling is complex [11–13]. According to one model, virulent strains of Mtb are capable of inhibiting host cell apoptosis during the early phase of the infection to allow for intracellular replication but the bacteria induce necrosis in order to exit the host cell at a later stage [14]. The discovery of *Mtb* genes that inhibit host cell apoptosis such as *nuoG* [15], *pknE* [16], *secA2* [17], *Rv3654c* [18], and *ndk* [19] supports this model. Furthermore, the Mtb *nuoG* mutant is attenuated in the mouse model of tuberculosis, thus illustrating the importance of host cell apoptosis inhibition for Mtb virulence [15]. Consistently, mice with reduced host cell apoptosis induction upon Mtb infection are more susceptible [20]. The mechanisms leading to increased host resistance include an increase in efferocytosis of apoptotic host cells leading to killing of the bacteria [21,22]. In addition, there are various lines of evidence that increased host cell apoptosis will lead to a more rapid and increased cytolytic T-cell response [17,23,24].

In contrast to apoptosis, host cell necrosis induction is associated with increased host susceptibility and virulence of Mtb as well as *Mycobacterium marinum* (Mm) in mice and in zebrafish [20,25]. Several studies demonstrated the central role of host cell eicosanoids lipoxin A_4_ (LXA_4_) and prostaglandin E_2_ (PGE_2_) in the regulation of host cell apoptosis versus necrosis induction and their importance for bacterial virulence and host resistance [24,26,27]. The enzyme Leukotriene A4 hydrolase (LTA4H) regulates synthesis of the eicosanoids LXA_4_ and leukotriene B_4_ (LTB_4_); excessive production of either lipid mediator leads to macrophage necrosis [28]. Polymorphisms in LTA4H in humans are associated with hypersusceptibility to mycobacterial infections [29]. Lysosomal destabilization and macrophage necrosis was found to occur following accumulation of about 20 or more intracellular bacteria [30,31]. The escape of Mtb from the phagosome to the cytosol precedes necrosis and exit from the host cell [32–34]. The Mtb type VII secretion systems, ESX-1 and ESX-5, are implicated in host cell necrosis induction. The deletion of the Mtb ESX-1 secretion system leads to a reduced induction of host cell necrosis and dissemination of the mutant mycobacteria [35–37], this could be due the inability of mutant strains to escape from the phagosome [33,34,38]. The Mtb ESX-5 system is involved in mediating cell necrosis after the bacteria have escaped the phagosome [39]. The PE25/PP41 complex secreted via ESX-5 may be one of the effectors of ESX-5-mediated host cell necrosis as addition of the purified protein complex induced necrosis of macrophages [40].

Host cell necrosis induction by Mtb is important for cell exit and dissemination but the molecular mechanisms involved are still poorly understood. Here we describe the discovery of a tetracycline repressor family protein, Rv3167c, which negatively regulates the capacity of the bacteria to induce host cell necrosis. Infection of macrophage with the *Rv3167c* deletion strain (MtbΔ*Rv3167c*) led to a rapid increase in host cell necrosis via a novel host cell signaling pathway that involves the reduced activation of the protein kinase Akt leading to an increase in mitochondrial reactive oxygen species (mROS). Interestingly, we discovered that MtbΔ*Rv3167c* escape the phagosome in higher numbers than wild-type Mtb, which most likely triggers the host cell necrosis signaling. Finally, aerosol infection of mice demonstrated the increased virulence of MtbΔ*Rv3167c*. In conclusion, we find that Rv3167c regulates the escape of Mtb from the phagosome, which marks the beginning of the host cell exit program of the Mtb intracellular life cycle.

## Results

### Rv3167c is important for inhibition of host cell programmed necrosis

**Fig 1:**
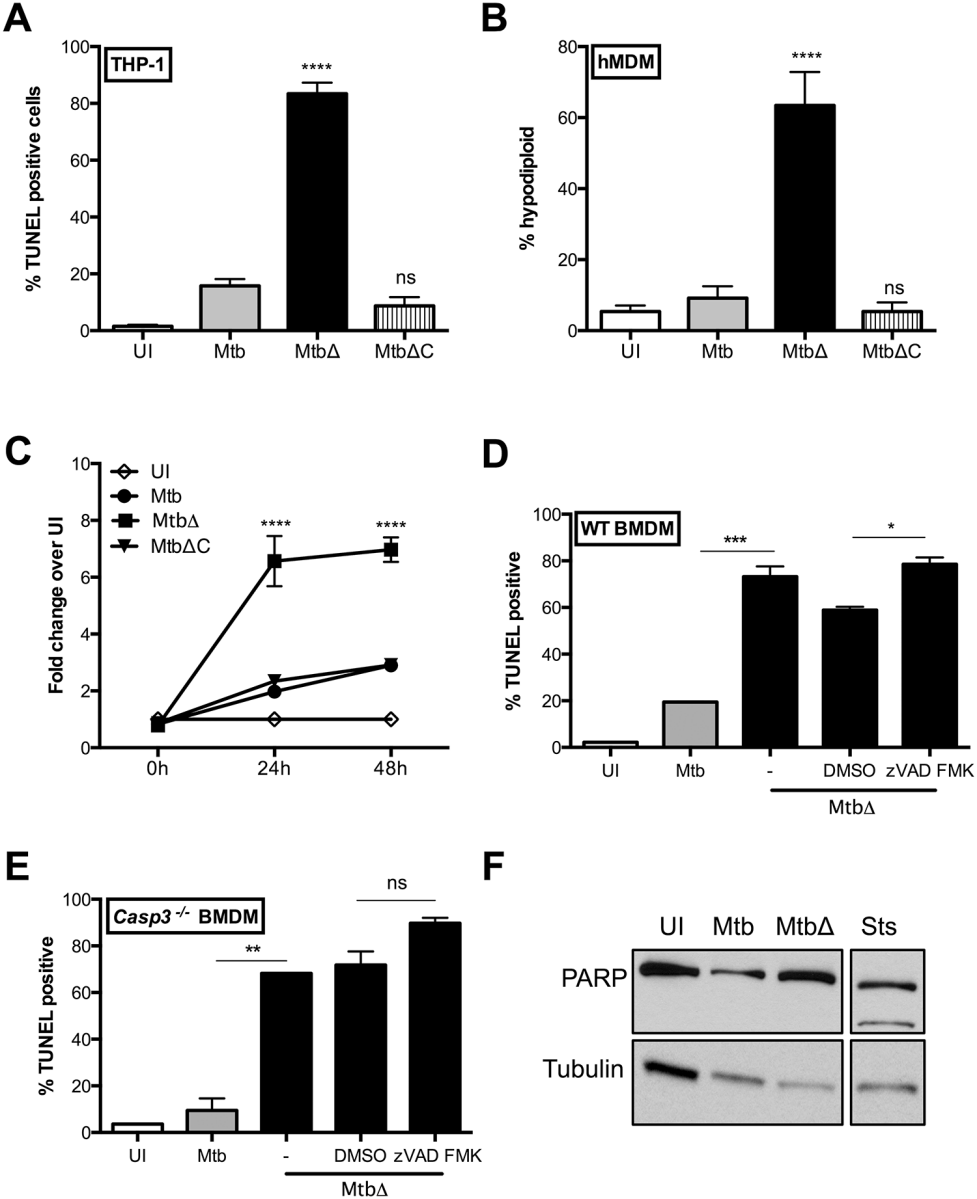
Mtb Rv3167c is important for inhibition of host cell death. Cell death induction in uninfected (UI), wild-type Mtb (Mtb), *Rv3167c* deletion mutant Mtb (MtbΔ) or complemented mutant Mtb (MtbΔC) bacteria infected cells was determined. (A) TUNEL staining and flow cytometry of THP1 cells at 24h (mean ± S.E.M, n = 6). (B) Cell death induction in human monocyte-derived macrophages (hMDMs) at 48h was measured by hypodiploid staining and flow cytometry. Cells were obtained from two independent donors (mean ± S.E.M, n = 6). (C) Release of adenylate kinase into supernatants from uninfected and infected THP1 cells was measured by Toxilight assay at the indicated time points (mean ± S.E.M, n = 6). Cell death induction in (D) wild type (WT) and (E) *Casp3* ^*-/-*^ BMDMs was determined by TUNEL staining and flow cytometry at 24h. Cells were treated with the pan caspase inhibitor zVAD-fmk (40μM) for one hour prior to and throughout the infection (mean ± S.E.M, n = 3). (F) PARP cleavage in THP1 cells at 24h detected by western blotting of whole cell lysates obtained from infected cells or after treatment with apoptosis inducer staurosporine (Sts). Image is representative of three independent experiments.

We previously performed a gain-of-function genetic screen and identified a genomic region in Mtb H37Rv containing anti-apoptotic genes (S1A Fig) [15]. A series of deletion mutants spanning several genes within this region was generated and tested for loss of apoptosis inhibition. THP1 cells were infected with wild-type Mtb (Mtb) and the deletion mutants and stained for genomic DNA fragmentation using TUNEL assay. Two deletion mutants, one being the single gene *nuoG* mutant [15], and the other a five gene deletion mutant designated 7/10, induced higher levels of cell death compared to the Mtb control (S1B Fig). Screening of genes within the 7/10 region revealed that deletion of *Rv3167c* had the maximal effect on loss of cell death inhibition. Infection with the deletion mutant MtbΔ*Rv3167c* (MtbΔ) resulted in almost a 3-fold increase in TUNEL-positive THP1 cells compared to infection with the control Mtb strain (S1C Fig). Both southern blotting and RT-PCR confirmed deletion of *Rv3167c* (S2A–S2C Fig). Increased cell death induction by MtbΔ*Rv3167c* was also observed in primary human monocyte derived macrophages (hMDMs) by hypodiploid staining which measures loss of genomic DNA content following cell death (Fig 1B). Cell death induction by the complement strain MtbΔ*Rv3167c*-C (MtbΔC) was comparable to Mtb (Fig 1A and 1B), thus confirming that *Rv3167c* is required for Mtb-mediated host cell death inhibition. Replication of MtbΔ is similar to Mtb; both, in infected THP1 cells and in growth media (S2D and S2E Fig). Rv3167c is most likely a member of the tetracycline-like family of regulators (TFR) since 89% of the Rv3167c amino acid sequence can be modeled with 99.9% confidence to the highest scoring template, the TFR SCO0332 of *Streptomyces coelicolor*, using Phyre2 software.

Although TUNEL staining has been historically used for detection of apoptotic DNA fragmentation, recent studies have shown that necrotic cells can also be TUNEL positive [41–43]. A characteristic feature of apoptotic cells is preservation of cell membrane integrity [3]. To determine whether cell death induced by MtbΔ*Rv3167c* is accompanied by cell membrane damage, we tested for the presence of adenylate kinase, normally located within healthy cells, in the supernatants of THP1 cells using the Toxilight assay. At 24 and 48h, a 3-fold higher level of adenylate kinase activity was detected in supernatant from MtbΔ*Rv3167c*-infected cells compared to uninfected controls (Fig 1C). Mtb-infected cells also undergo necrosis albeit at lower levels compared to MtbΔ*Rv3167c*-infected cells. Mtb has been previously shown to induce necrosis in a dose and time dependent manner and our data supports this observation [44]. It is important to note that the Toxilight assay cannot differentiate between primary necrosis or secondary necrosis of apoptotic cells and consequently further analysis into the nature of the induced cell death was required.

Execution of apoptotic cell death requires the cleavage and activation of the effector caspases-3, -6 and -7 [2]. We infected wild type (WT) and *Casp3* ^*-/-*^ bone marrow derived macrophages (BMDMs) and performed TUNEL staining to confirm that MtbΔ*Rv3167c* does not induce apoptotic cell death. No differences in TUNEL-positive cells were observed between MtbΔ*Rv3167c*-infected WT and *Casp3* ^*-/-*^ BMDMs (Fig 1D and 1E). To ensure that the lack of death inhibition in *Casp3* ^*-/-*^ BMDMs is not due to redundancy of caspase-3 with other effector caspases, the pan-caspase inhibitor zVAD-FMK was added to MtbΔ*Rv3167c*-infected cells. Inclusion of zVAD-FMK did not inhibit MtbΔ*Rv3167c*-induced cell death in both WT and *Casp3* ^*-/-*^ BMDMs (Fig 1E) although it did inhibit apoptosis induced by camptothecin (S3A Fig). We also did not observe cleavage of the DNA repair enzyme PARP, another feature of apoptosis, in Mtb or MtbΔ*Rv3167c*-infected THP1 cells (Fig 1F). Furthermore, the zVAD-FMK inhibitor was added to infected *Ripk3* ^*-/-*^ cells and had no effect on MtbΔ*Rv3167c*-induced cell death (S3D Fig). These results show that *Rv3167c* is required for inhibition of Mtb-induced necrotic host cell death.

### MtbΔ*Rv3167c*-mediated cell death involves lysosomal membrane permeabilization

**Fig 2:**
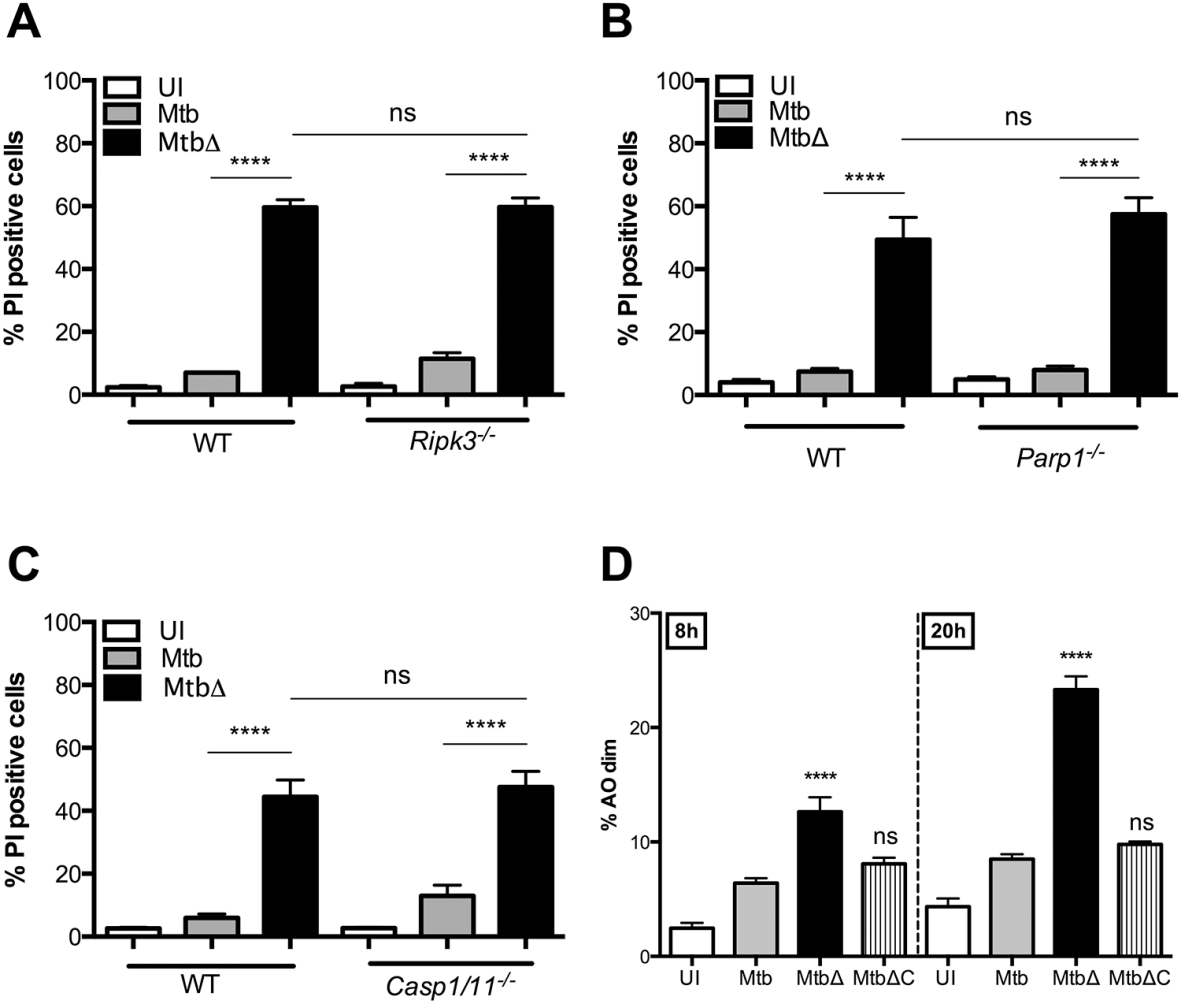
MtbΔ*Rv3167c* mediates cell death via programmed necrosis. (A) Necrosis induction in WT and *Ripk3* ^*-/*-^ BMDMs was measured by PI staining and flow cytometry at 24h (mean ± S.E.M, n = 3) in uninfected (UI), wild-type Mtb (Mtb), *Rv3167c* deletion mutant Mtb (MtbΔ) or complemented mutant Mtb (MtbΔC) bacteria infected cells. (B) Necrosis induction in WT and *Parp1* ^*-/-*^ BMDMs at 72h was determined by PI staining (mean ± S.E.M, n = 3). (C) Necrosis induction in WT and *Casp1/11* ^*-/-*^ BMDMs was determined by PI staining at 48h (mean ± S.E.M, n = 6). (D) Increases in lysosomal permeabilization were measured by acridine orange staining at indicated time points (mean ± S.E.M, n = 6).

Next, we investigated the involvement of programmed necrosis pathways in cell death mediated by MtbΔ*Rv3167c*. In conditions where caspase-8 expression and activation are inhibited, the serine threonine protein kinases RIPK1 and RIPK3 induce necrosis downstream of TNF-receptor ligation via increased reactive oxygen species (ROS) generation, mitochondrial fission and formation of plasma membrane pores [45–48]. RIPK1 and RIPK3 have also been implicated in TNF-mediated necrosis in Mm-infected zebrafish [49]. We investigated the involvement of RIPK1 and RIPK3 in MtbΔ*Rv3167c*-induced cell death by using *Ripk3* ^*-/-*^ BMDM’s and the RIPK1 inhibitor necrostatin1 (Nec1). Similar levels of PI-positive cells were observed in MtbΔ*Rv3167c*-infected *Ripk3* ^*-/-*^ BMDMs and Nec1 treated cells compared to WT BMDMs and solvent control-treated cells respectively (Figs 2A and S3B). Nec1 efficacy was confirmed by its ability to inhibit LPS and zVAD FMK induced RIPKI dependent cell death (S3C Fig) [50]. Necrosis induction following TNF treatment has been reported to change to apoptosis in the absence of RIPK1 and consequently, the absence of an effect on cell death induction by MtbΔ*Rv3167c* could be due to an increase in apoptosis in cells deficient in RIPK1/3 signaling [1]. Addition of zVAD-FMK to *Ripk3* ^*-/-*^ cells did not affect MtbΔ*Rv3167c*-induced cell death thereby ruling out a switch between apoptosis and necrosis in MtbΔ*Rv3167c*-infected cells (S3D Fig). Infection of *Tnfr1* ^-/-^ BMDMs established that MtbΔ*Rv3167c*-induced necrosis was independent of TNF signaling (S4A Fig). The DNA repair enzyme PARP1 has been implicated in necrosis induction via ATP depletion and nuclear translocation of mitochondrial apoptosis inducing factor in response to DNA alkylating agents and infection with BCG and enterovirus71 [51–53]. Necrosis induction by MtbΔ*Rv3167c* is independent of PARP1 since similar levels of necrosis were observed in infected *Parp1* ^*-/-*^ BMDMs and WT control cells (Fig 2B). The pro-inflammatory caspases, caspase-1 and caspase-11 have been shown to be involved in necrosis induction in response to several bacterial pathogens [54,55]. The role of these caspases in MtbΔ*Rv3167c*-induced necrosis was excluded by PI staining of *Casp1/11* ^*-/-*^ BMDMs (Fig 2C). NLRP3-dependent but caspase-1-independent necrosis has been reported to occur in response to infection with Mtb and *Shigella flexneri* [56,57]. Using immortalized NLRP3-deficient BMDMs we ruled out involvement of NLRP3 in MtbΔ*Rv3167c*-induced necrosis (S4C Fig). Silencing of the inflammasome component ASC did not inhibit MtbΔ*Rv3167c*-induced necrosis as measured by the toxilight assay, rather necrosis was increased in MtbΔ*Rv3167c*-infected THP1shASC cells compared to control cells (S4D Fig). Involvement of IFNβ signaling (S4E and S4F Fig) and TLR signaling (S5A–S5C Fig) was also ruled out in necrosis induction by MtbΔ*Rv3167c*. These data indicate that MtbΔ*Rv3167c* does not engage the pathways of programmed necrosis currently described in the literature to induce host cell death.

Lysosomal membrane permeabilization (LMP) and the subsequent release of lysosomal contents into the cell cytosol leads to cell death [58,59]. Moderate lysosomal permeabilization leads to apoptosis while more severe damage precedes necrosis [60]. Lysosomal permeabilization and cathepsin release into the cytosol has been previously observed in cells infected with Mtb at high MOI [61]. To investigate whether LMP contributes to MtbΔ*Rv3167c*-induced cell death, we used the dye acridine orange (AO) that accumulates within lysosomes. A two-fold increase in the number of cells with loss of AO staining was observed as early as 8h post infection in MtbΔ*Rv3167c*-infected THP1 cells compared to Mtb-infected controls (Fig 2D). After 20h of infection the difference was even more pronounced with only about 8% of Mtb-infected cells showing low AO-staining compared to about 25% in mutant infected cells (Fig 2D). This indicates that LMP precedes necrosis induction by MtbΔ*Rv3167c* and is not merely a consequence of the disintegration of the cell triggered via a different mechanism.

### MtbΔ*Rv3167c* causes increased macroautophagy but not autophagic cell death

**Fig 3:**
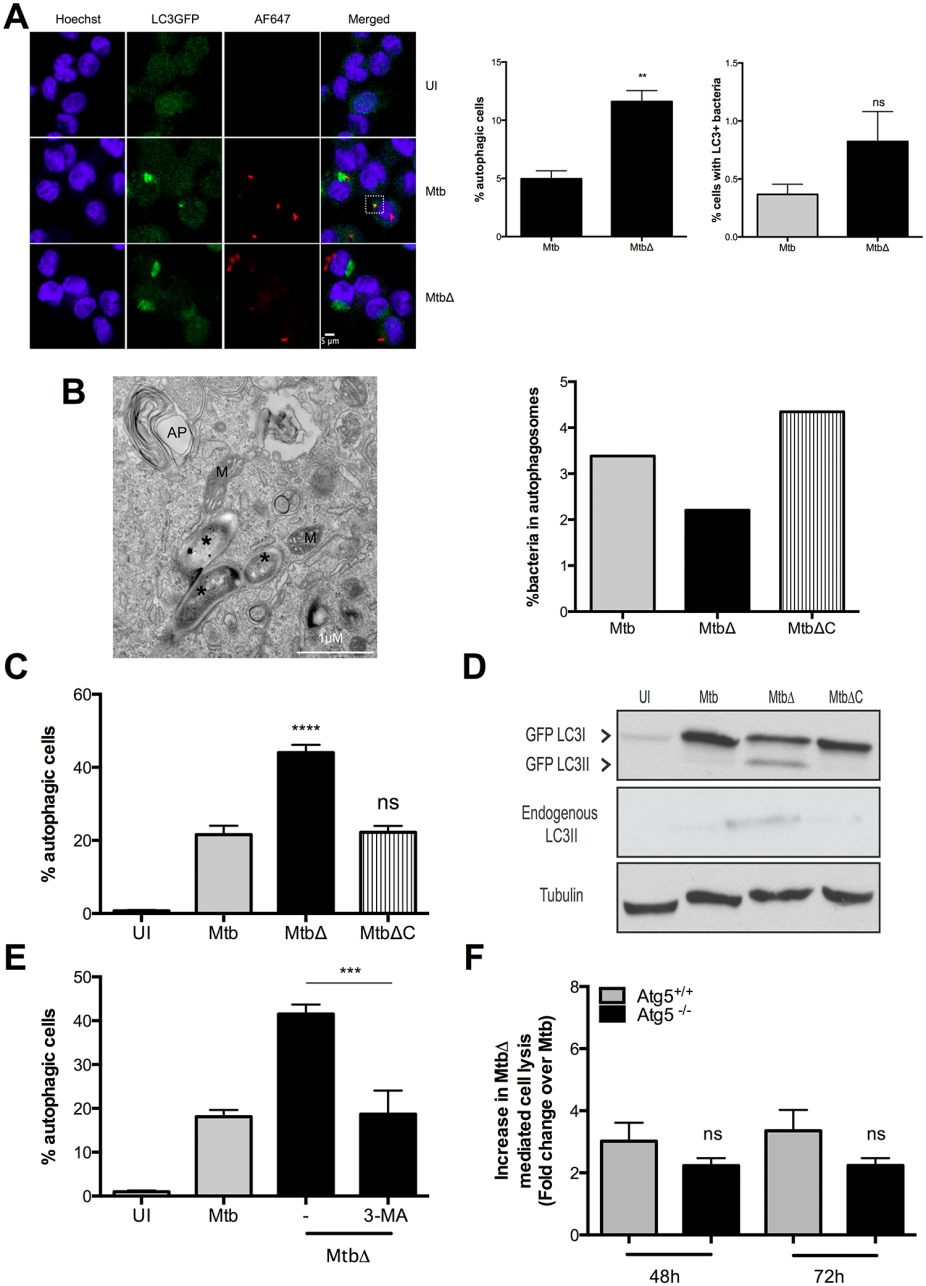
MtbΔ*Rv3167c* causes increased macroautophagy but not autophagic cell death. Uninfected (UI), wild-type Mtb (Mtb), *Rv3167c* deletion mutant Mtb (MtbΔ) or complemented mutant Mtb (MtbΔC) bacteria infected cells were analyzed as described. (A) Autophagy induction by AF647-NHS ester stained bacteria in THP1 LC3GFP cells at 8h examined by confocal microscopy. *Left*: Representative fluorescent image. The white box indicates bacteria colocalizing with LC3GFP. *Right*: Quantitative analysis of autophagic cells and cells with LC3+ bacteria. At least 450 cells were counted per condition per experiment. Statistical analysis was performed using unpaired Student’s t test (mean ± S.E.M, n = 3). (B) *Left*: Representative TEM image showing absence of colocalization between bacteria and autophagosomes at 24h. *—bacteria; AP—autophagosome; M—mitochondria. *Right*: Quantitative analysis of bacteria within autophagosomes as observed by TEM. Atleast 10 cells were counted per condition per experiment (n = 2). (C) Autophagy induction in THP1 LC3GFP cells measured at 16h by flowcytometry (mean ± S.E.M, n = 6). (D) Conversion of GFP tagged and endogenous LC3I to LC3II detected by western blotting in whole cell lysates. Image is representative of three independent experiments. (E) Inhibition of MtbΔ*Rv3167c*-induced autophagy in presence of 3-MA (5mM) measured by flowcytometry at 16h (mean ± S.E.M, n = 4). (F) Necrosis induction in autophagy competent (Atg5^+/+^) and autophagy deficient (Atg5^-/-^) BMDMs measured using Toxilight assay (mean ± S.E.M, n = 6).

Autophagy is a catabolic process that allows for cell survival via recycling of cellular contents and contributes to pathogen elimination [62]. However, autophagy induction can also lead to cell death [63]. Therefore, we investigated whether MtbΔ*Rv3167c* could induce autophagy in macrophages. First, we analyzed recruitment of LC3 into aggregates, an indicator of autophagy, by confocal microscopy [64]. THP1 cells expressing GFP-tagged LC3 (THP1 LC3GFP) were infected with AF647-NHS stained bacteria and at 8h, the percentage of cells showing aggregation of LC3 was estimated. A two-fold increase in autophagosome formation was observed in MtbΔ*Rv3167c*-infected cells compared to Mtb-infected control cells (Fig 3A). Previous studies have shown that Mtb induces xenophagy resulting in co-localization of bacteria with autophagosomes and bacterial killing [65,66] However, we observed minimal colocalization of both Mtb and MtbΔ*Rv3167c* (<1%) with autophagosomes (Fig 3A). This was confirmed by examination of infected THP1 cells by transmission electron microscopy (TEM) (Fig 3B). Next, we measured conversion of cytosolic LC3I to autophagosomal membrane bound LC3II, another hallmark of autophagy [64]. Uninfected and infected THP1 LC3GFP cells were washed with saponin-containing buffer leading to removal of cytosolic LC3I-GFP. Retention of autophagosomal membrane-bound LC3II-GFP was examined by flow cytometry [67]. A two-fold increase in autophagy induction was observed in MtbΔ*Rv3167c*-infected cells compared to those infected with Mtb and MtbΔ*Rv3167c*-C (Fig 3C). Increased conversion of both GFP tagged and endogenous LC3I to LC3II in MtbΔ*Rv3167c*-infected cells was also seen by immunoblotting (Fig 3D). Autophagy induction by MtbΔ*Rv3167c* was confirmed using 3-methyladenine (3-MA), a classical autophagy inhibitor [64]. Inclusion of 3-MA inhibited LC3II formation by MtbΔ*Rv3167c* in THP1 LC3GFP cells (Fig 3E).

Increased accumulation of LC3II can be attributed either to an increase in autophagosome formation or to a decrease in LC3II degradation due to inhibition of autophagosome-lysosome fusion and maturation [64]. Addition of the vacuolar H^+^ ATPase inhibitor bafilomycin A1 (BafA1) that inhibits autophagosomal degradation to MtbΔ*Rv3167c*-infected THP1 LC3GFP cells led to a further increase in LC3II levels (S6A Fig). Autophagosome maturation leads to the degradation of LC3GFP to yield free GFP [64]. GFP was detected only in MtbΔ*Rv3167c*-infected cells by immunoblotting (S6B Fig). These data indicate that MtbΔ*Rv3167c*-induced autophagy but did not inhibit the maturation of the autophagosome. Finally to determine whether necrotic death of MtbΔ*Rv3167c*-infected cells was a consequence of autophagy induction, we measured adenylate kinase release from Atg5^fl/fl^ LysM Cre^+^ (Atg5^-/-^) and Atg5^fl/fl^ LysM Cre^-^ (Atg5^+/+^) BMDMs. We observed no differences in necrosis induction by MtbΔ*Rv3167c* in autophagy-deficient Atg5^-/-^ cells compared to the Atg5^+/+^ controls (Fig 3F). Additionally, the inclusion of 3-MA did not result in inhibition of MtbΔ*Rv3167c*-induced cell death in THP1 cells (S6C Fig). Therefore, while MtbΔ*Rv3167c*-infected cells undergo macroautophagy, this does not contribute to their death via necrosis.

### Rv3167c regulates escape of Mtb from the phagosome

**Fig 4:**
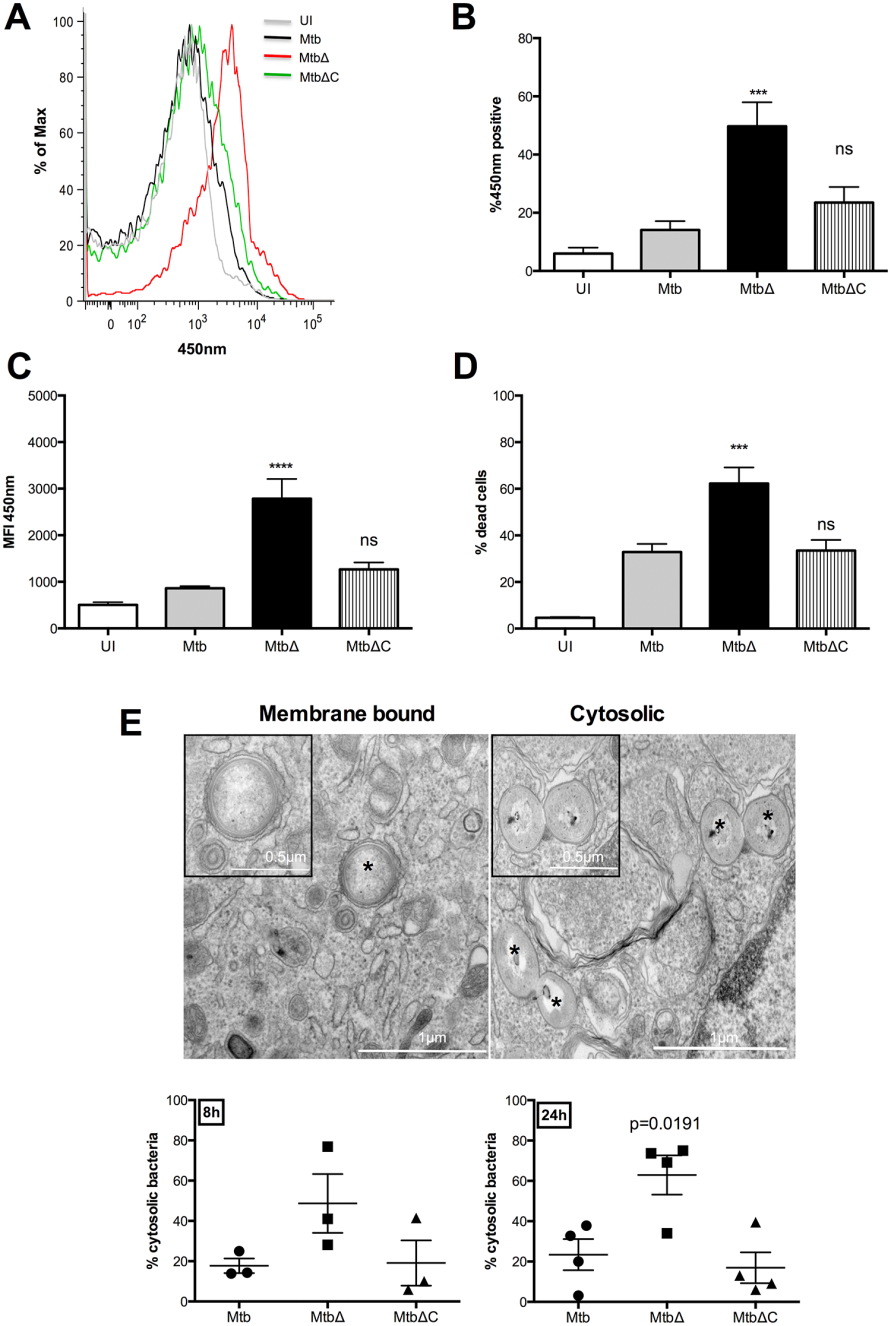
Rv3167c regulates escape of Mtb from the phagosome. Uninfected (UI), wild-type Mtb (Mtb), *Rv3167c* deletion mutant Mtb (MtbΔ) or complemented mutant Mtb (MtbΔC) bacteria infected cells were analyzed as described. CCF4 FRET-based flow cytometry was performed in combination with Live-Dead cell viability assays to examine phagosomal escape. Cells were gated to exclude dead populations and subsequent analysis was performed on live cells only. (A) Representative histograms demonstrating increased loss of FRET at 48h (corresponding to increased MFI_450nm_) in MtbΔ-infected THP1 cells compared to Mtb- and MtbΔC-infected cells. (B) and (C) Quantification of (A) (mean ± S.E.M, n = 6). (D) Cell death induction measured by Live-Dead cell viability assay (mean ± S.E.M, n = 6). (E) *Above*: Representative TEM image of membrane bound and cytosolic mycobacteria within THP1 cells. *—bacteria. Inset: Magnified images of bacteria. *Below*: Quantitative analysis of cytosolic bacteria as observed by TEM at 8h and 24h. At least 10 cells were counted per condition per experiment (mean ± S.E.M, n = 3).

The concept that Mtb resides within phagosomal compartments at all times has been challenged by recent studies demonstrating bacillary escape to the cytosol both *ex vivo* and *in vivo* [32–34,38]. Necrosis induction by Mtb and Mm was shown to closely follow escape to the cytosol [33,68]. We examined cytosolic escape by MtbΔ*Rv3167c* using a fluorescence resonance energy transfer (FRET) based assay [33,34,69]. Uninfected and infected THP1 cells differentiated for three days with PMA were loaded with CCF4-AM. Intact CCF4-AM emits green fluorescence (535nm) due to FRET between the fluorescent moieties. Cleavage of CCF4-AM by β-lactamase expressed by cytosolic bacteria leads to FRET loss and a shift in the emission wavelength to 450nm that was measured by flow cytometry. Cells were co-stained with Live/Dead Fixable Red stain to restrict analysis to live cells only. While minor increases in fluorescence emission at 450nm were observed in Mtb-infected cells compared to uninfected cells at 48h, the largest shift in the CCF4 emission spectrum was seen in MtbΔ*Rv3167c*-infected cells (Fig 4A and 4B). A three-fold increase was observed in MFI_450nm_ of cells infected with MtbΔ*Rv3167c* compared to Mtb-infected controls. Increased cytosolic escape of MtbΔ*Rv3167c* was reversed following complementation (Fig 4A–4C). The pro-necrotic phenotype of MtbΔ*Rv3167c* was preserved in these macrophages (Fig 4D). Bacterial β-lactamase activity was not affected by either deletion of *Rv3167c* or gene complementation (S7 Fig).

To confirm the increased cytosolic escape by MtbΔ*Rv3167c*, we examined infected THP1 cells by TEM and quantified cytosolic bacteria in a double-blinded fashion by examining for absence of phagosomal membranes in healthy cells (Fig 4E, *above*). Increased presence of MtbΔ*Rv3167c* was observed in the cytosol at 8h compared to controls although statistical significance was not achieved. At 24h, 60% of MtbΔ*Rv3167c* were found to be cytosolic compared to 20% of Mtb corroborating the increased cytosolic escape by MtbΔ*Rv3167c* observed with the CCF4-AM assay (Fig 4E, *below*). A reduction of cytosolic escape was observed in cells infected with the complemented *MtbΔRv3167c*-C strain (Fig 4E, *below*). These data suggest that Rv3167c negatively regulates Mtb escape from the phagosome to the cytosol, an event that has been shown to be followed by induction of host cell necrosis [33,34].

### Host kinase Akt1/2 is central to autophagy and necrosis induction by MtbΔ*Rv3167c*

**Fig 5:**
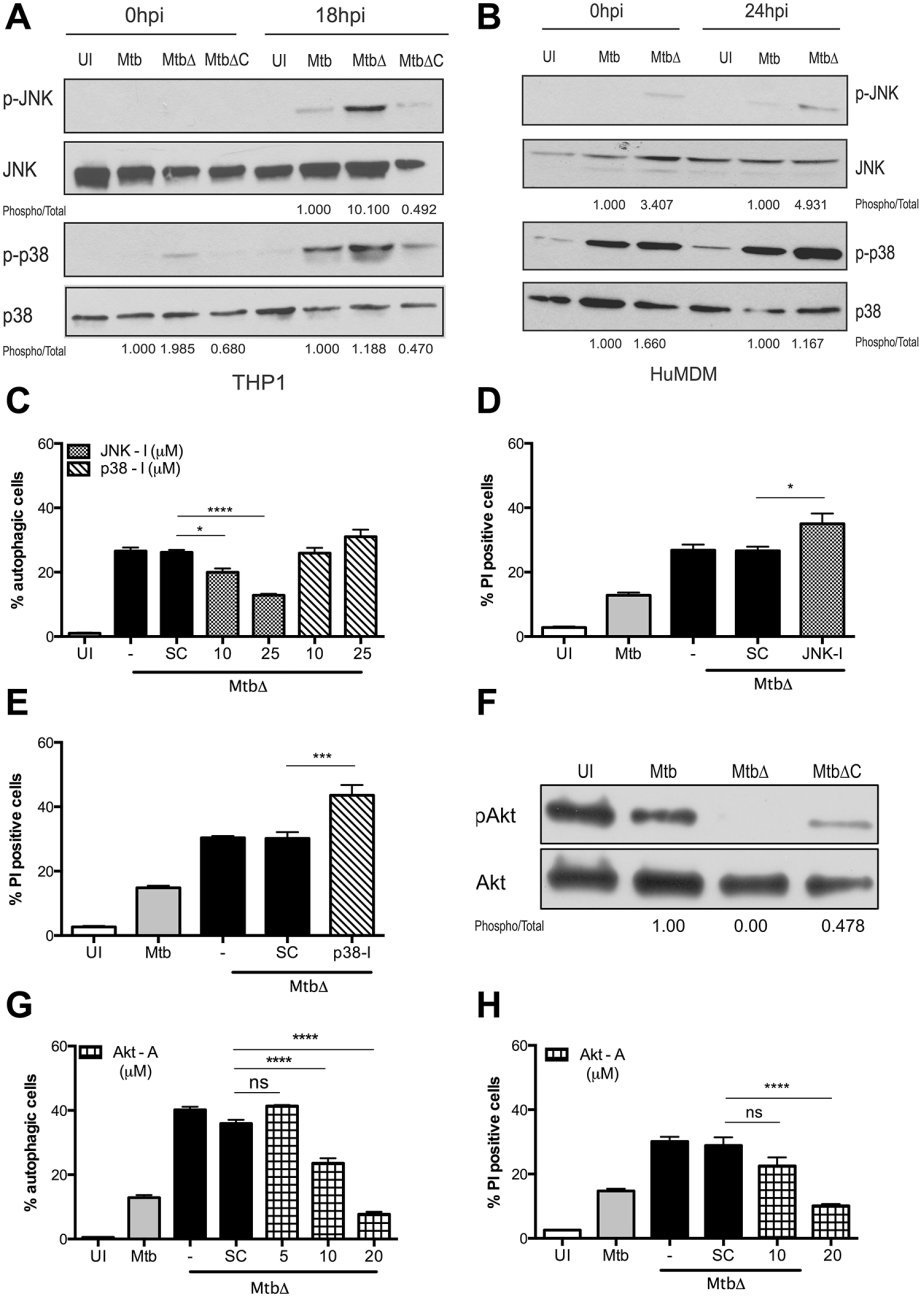
Host kinase Akt1/2 regulates MtbΔ*Rv3167c*-mediated autophagy and necrosis signaling. Uninfected (UI), wild-type Mtb (Mtb), *Rv3167c* deletion mutant Mtb (MtbΔ) or complemented mutant Mtb (MtbΔC) bacteria infected cells were analyzed as described. (A) JNK and p38 MAPK phosphorylation in THP1 LC3GFP cells at 18h detected by western blotting of whole cell lysates. Image is representative of three independent experiments. (B) JNK and p38 MAPK phosphorylation in human monocyte derived macrophages (hMDMs) at 24h detected by western blotting of whole cell lysates. Image is representative of three independent experiments. Cells were obtained from two donors. (C) Autophagy induction in presence of JNK (JNK-I—SP600125) and p38 MAPK (p38-I-SB203580) inhibitors detected by flow cytometry (mean ± S.E.M, n = 6). (D) Effect of JNK inhibitor (JNK-I-SP600125, 25μM) and (E) p38MAPK inhibitor (p38-I-SB203580, 25μM) on necrosis measured by PI staining and flow cytometry at 24h (mean ± S.E.M, n = 6). (F) Akt phosphorylation in THP1 LC3GFP cells detected at 16h by western blotting. Image is representative of three independent experiments. (G) Autophagy induction in presence of Akt activator (Akt—A—sc-79) examined by flow cytometry at 16h (mean ± S.E.M, n = 8). (H) Necrosis induction in presence of Akt activator (Akt—A—sc-79) in THP1 macrophages measured by flow cytometry at 24h (means ± S.E.M, n = 6). For immunoblots, numbers below indicate fold change in band intensity compared to Mtb sample after normalization to loading control.

Next we investigated the molecular mechanisms underlying autophagy induction by MtbΔ*Rv3167c*. The mitogen activated protein kinases (MAPKs) JNK and p38 have been implicated in autophagic responses of cells infected with Mtb following exposure to cytokines and vitamin D3 [70,71]. Mtb Eis has been shown to inhibit autophagy induction by suppressing JNK activation [72]. Hence we examined MAPK activation in response to MtbΔ*Rv3167c* infection by immunoblotting for phosphorylated forms in whole cell lysates prepared from infected THP1 LC3GFP cells at the indicated times. JNK activation was not observed at 0h, however increased JNK phosphorylation was detected in MtbΔ*Rv3167c*-infected cells compared to those infected with Mtb and MtbΔ*Rv3167c*-C at 18h (Fig 5A). In contrast to JNK, increased p38MAPK activation was observed in MtbΔ*Rv3167c*-infected cells at 0h. However by 18h, p38MAPK phosphorylation in MtbΔ*Rv3167c*-infected cells was similar to control cells infected with Mtb and MtbΔ*Rv3167c*-C (Fig 5A). Consistent results were observed in human monocyte-derived macrophages (hMDMs) as well; however, elevated JNK activation could be detected earlier at 0h in MtbΔ*Rv3167c*-infected cells (Fig 5B). We then determined whether JNK and p38MAPK contributed to autophagy induction by MtbΔ*Rv3167c*. THP1 LC3GFP cells were pre-treated and infected with JNK (SP600125) or p38MAPK (SB203580) inhibitors prior to infection with MtbΔ*Rv3167c*; the percentage of autophagic cells was measured by flow cytometry. Inclusion of the JNK inhibitor led to a partial, dose-dependent decrease in autophagy induction by MtbΔ*Rv3167c* while the p38MAPK inhibitor exerted no effects (Fig 5C). Neither of the inhibitors reversed the pro-necrotic phenotype of MtbΔ*Rv3167c*, instead a modest increase in PI-positive cells was observed in both cases (Fig 5D and 5E).

The serine threonine protein kinase Akt functions as a critical negative regulator of autophagy at the initiation stage by activating mTOR and at the nucleation step by phosphorylating Beclin1 [73,74]. Inhibition of Akt activation has been implicated both in macroautophagy induction in response to nutritional stresses as well as selective autophagy-induction in response to pathogens such as *Toxoplasma gondii* and *Salmonella typhimurium* [73,75,76]. To determine role of Akt in autophagy-induction by MtbΔ*Rv3167c*, we examined Akt phosphorylation by immunoblotting. A complete loss of Akt activation was observed in MtbΔ*Rv3167c*-infected cells compared to the controls (Fig 5F). Consistently, the Akt activator, sc-79, inhibited autophagy induction by MtbΔ*Rv3167c* in a dose-dependent manner (Fig 5G) [77]. Additionally, Akt inhibition exerts effects on MtbΔ*Rv3167c*-mediated necrosis as well, since sc-79 significantly reduced MtbΔ*Rv3167c*-induced necrotic cell death (Fig 5H).

### Mitochondrial ROS is required for cell death and autophagy induction by MtbΔ*Rv3167c*

**Fig 6:**
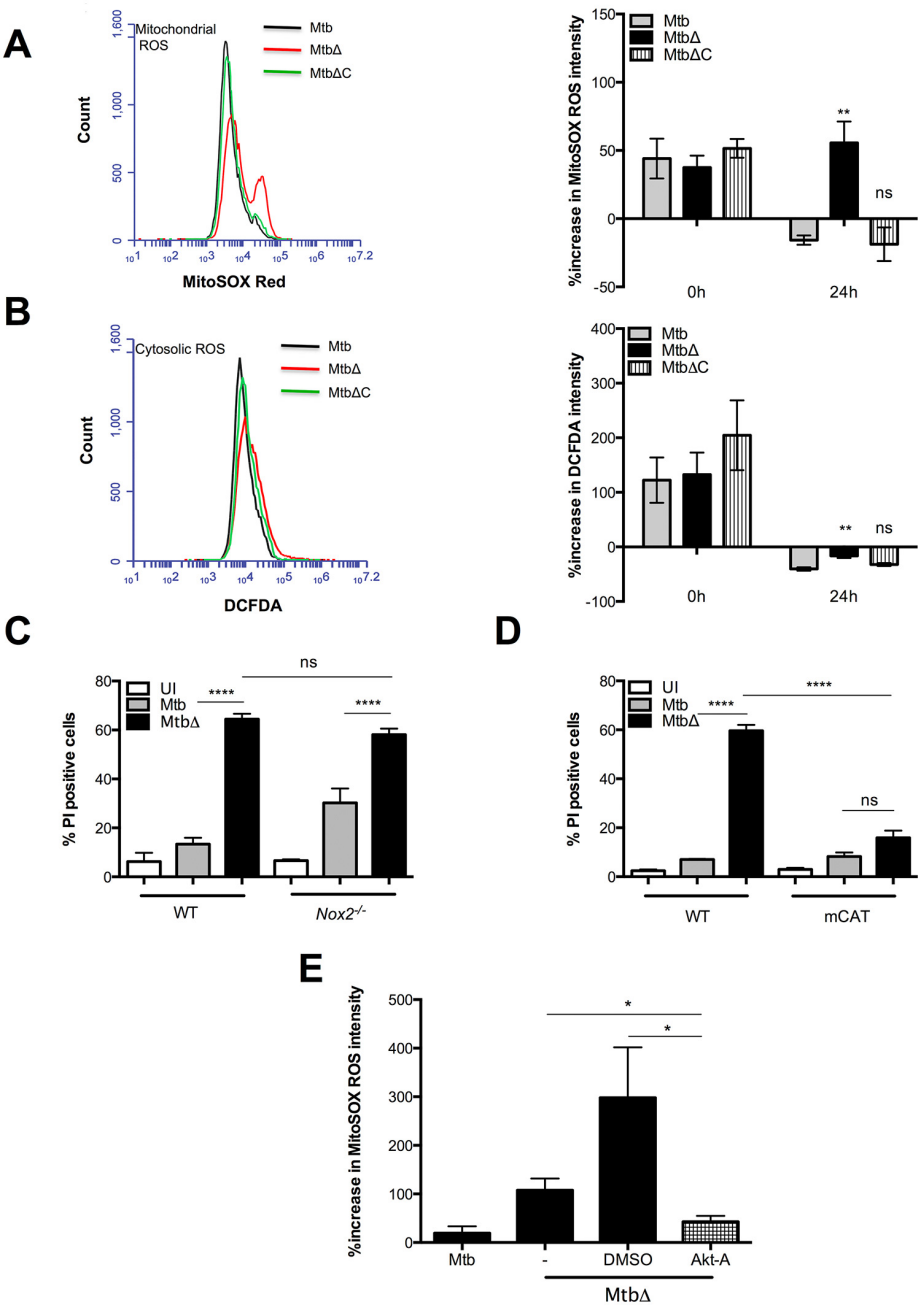
Mitochondrial ROS is required for cell death and autophagy induction by MtbΔ*Rv3167c*. Uninfected (UI), wild-type Mtb (Mtb), *Rv3167c* deletion mutant Mtb (MtbΔ) or complemented mutant Mtb (MtbΔC) bacteria infected cells were analyzed as described. (A) *Left*: Representative histogram of mitochondrial ROS measurement using MitoSOX Red staining of BMDMs at 24h. *Right*: Quantification of mitochondrial ROS by estimating increase in MitoSOX Red fluorescence intensity (mean ± S.E.M, n = 4) (B) *Left*: Representative histogram of cellular ROS measurement using DCFDA staining of BMDMs at 24h. *Right*: Quantification of cellular ROS by estimating increase in DCFDA fluorescence intensity (means ± S.E.M, n = 4). For (A) and (B), percentage increase in mean fluorescent intensity of infected cells compared to UI cells was calculated after subtracting background fluorescence. (C) Necrosis induction in WT and *Nox2* ^*-/-*^ BMDMs was determined by PI staining and flow cytometry at 48h (mean ± S.E.M, n = 6). (D) Necrosis induction in BMDMs from WT and mitochondrial targeted catalase (mCAT) knock-in mice was determined by PI staining and flow cytometry at 24h (mean ± S.E.M, n = 3). (E) Effect of the Akt activator sc-79 on mitochondrial ROS was measured by staining with MitoSOX Red and flowcytometry (mean ± S.E.M, n = 3).

Mtb-mediated suppression of reactive oxygen species (ROS) generated by the host phagocytic NADPH oxidase complex (NOX2) has been shown to inhibit host cell apoptosis [78]. Conversely, necrosis-induction by Mm has been shown to require mitochondrial ROS generation [49]. To determine whether necrosis-induction by MtbΔ*Rv3167c* involves ROS, we first measured ROS levels in cells infected by MtbΔ*Rv3167c*. Uninfected and infected BMDMs were stained with the either 2’7’-dichlorofluorescein diacetate (DCFDA) or MitoSOX Red for measurement of cytosolic and mitochondrial ROS respectively. At 0h, similar levels of ROS were observed in all infected cells compared to the uninfected controls (Fig 6A and 6B). However by 24h, approximately three-fold higher levels of both cytosolic and mitochondrial ROS were detected in MtbΔ*Rv3167c*-infected cells compared to those infected with Mtb and MtbΔ*Rv3167c*-C (Fig 6A and 6B). Next we examined whether increased ROS levels contribute to necrosis induction by MtbΔ*Rv3167c* using the flavoprotein inhibitor diphenylene iodonium (DPI) and the ROS scavengers glutathione and N-acetyl cysteine (NAC). Inclusion of these inhibitors and scavengers reversed necrosis induction by MtbΔ*Rv3167c* to levels observed in uninfected cells (S8A and S8B Fig). Thus, elevated ROS levels in MtbΔ*Rv3167c*-infected cells contribute to their necrotic cell death. Furthermore, addition of DPI to MtbΔ*Rv3167c*-infected THP1 LC3GFP cells completely abrogated autophagy induction compared to control cells (S8C Fig).

As ROS in eukaryotic cells may be derived from the NOX2 complex or mitochondria, we sought to determine which of these sources is implicated in necrosis-induction by MtbΔ*Rv3167c*. Similar levels of necrosis induction by MtbΔ*Rv3167c* were detected in WT and *Nox2* ^*-/-*^ BMDMs by PI staining (Fig 6C). However, a complete inhibition of MtbΔ*Rv3167c*-mediated cell death was observed in mCAT BMDMs obtained from transgenic mice overexpressing mitochondrial targeted human catalase (Fig 6D). Increased mitochondrial ROS generation was also accompanied by a time dependent loss of mitochondrial membrane potential as measured by DIOC_6_ staining of MtbΔ*Rv3167c*-infected cells (S8D Fig). Increased mROS generation in MtbΔRv3167c-infected cells was found to be attributable to reduced Akt activation as inclusion of the Akt activator sc-79 inhibited mROS generation (Fig 6E). Taken together, our data reveal Akt and mitochondrial ROS to be critical regulators of MtbΔ*Rv3167c*-mediated necrosis and autophagy.

### Rv3167c regulates virulence of Mtb

**Fig 7:**
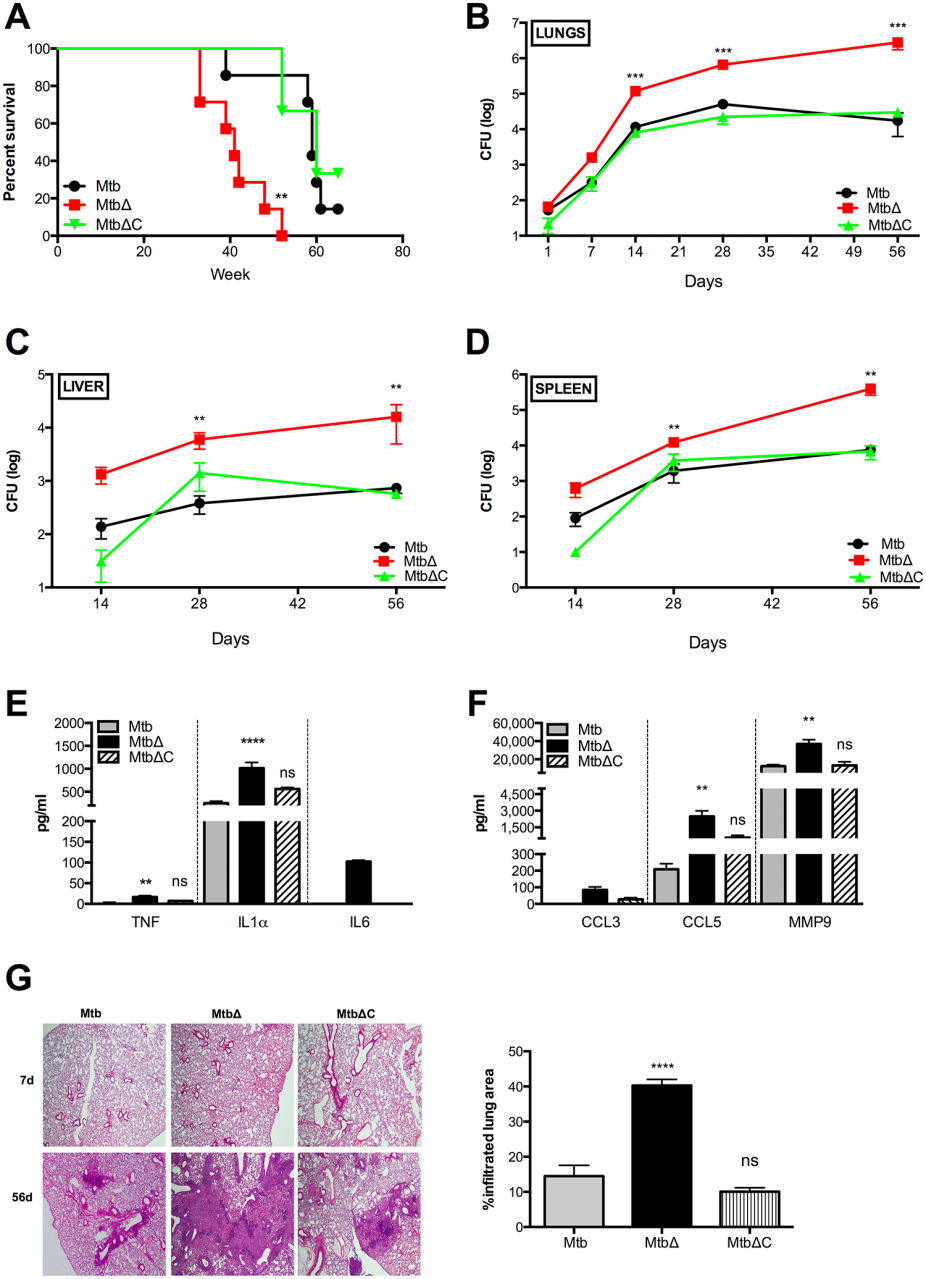
*Rv3167c* regulates virulence of Mtb. (A) Survival of C57Bl6 mice infected via the aerosol route with 100 CFU of wild-type Mtb (Mtb), *Rv3167c* deletion mutant Mtb (MtbΔ) or complemented mutant Mtb (MtbΔC) bacteria (n = 7 per experimental group) (B) Lung, (C) spleen and (D) liver bacterial burdens were determined at indicated times (mean ± S.E.M, n = 6) (E) Cytokines and (F) chemokines in lung tissue homogenates at 56d were quantified using a multiplex ELISA (mean ± S.E.M, n = 3). (G) *Left*: Representative image of H and E stained section of lung tissue. *Right*: Quantitative analysis of lung area exhibiting cellular infiltration at 56d performed using ImageJ (mean ± S.E.M, n = 3).

We assessed the contribution of *Rv3167c* to Mtb virulence *in vivo* by performing a survival study of C57Bl/6 mice infected with approximately 100 CFU of Mtb, MtbΔ*Rv3167c* and MtbΔ*Rv3167c*-C via the aerosol route. Increased mortality was observed in MtbΔ*Rv3167c*-infected mice (median survival time—33 weeks) compared to those infected with Mtb or MtbΔ*Rv3167c*-C (median survival times– 59 and 60 weeks respectively) (Fig 7A). Decreased survival following MtbΔ*Rv3167c* infection was also observed in immunodeficient SCID mice (S9A Fig). Lung bacterial burden on day one after infection was similar for all three strains indicating comparable initial inoculum of infection in both C57Bl/6 and SCID mice (Figs 7B and S9B). Relative to control mice, the lung bacillary burden was 10-fold higher in MtbΔ*Rv3167c*-infected animals at14 and 28 days, and this difference was magnified by day 56 following aerosol infection (Fig 7B). Increased bacterial burdens also were observed in the liver and spleen of MtbΔ*Rv3167c*-infected mice relative to control mice (Fig 7C and 7D). Higher levels of pro-inflammatory cytokines (TNF, IL1α and IL6) (Fig 7E) and chemokines (CCL3, CCL5 and MMP9) (Fig 7F) were detected in the lung tissues of mice infected with MtbΔ*Rv3167c*. Comparison of lung histopathology revealed a two-fold increase in cellular infiltration in MtbΔ*Rv3167c*-infected animals (Fig 7G).

Consistent with the findings in the mouse model of chronic TB infection, increased bacterial burdens were observed in the lungs of guinea pigs infected with *MtbΔRv3167c* relative to controls at 28 days following aerosol infection with similar bacterial loads (S9C and S9D Fig). MtbΔ*Rv3167c* was also found to induce cell death in *ex vivo* infection of guinea pig alveolar macrophages by TUNEL staining (S9E Fig). Collectively, these results show that Rv3167c negatively regulates Mtb virulence.

## Discussion

The intracellular location of bacteria has important consequences for their recognition by the host and the generation of innate and adaptive immune responses. Mtb was thought to restrict itself to a modified host cell phagosomal compartment after infection [79,80]. However, electron microscopy studies performed on infected macrophages and dendritic cells provided evidence that Mtb and other mycobacterial species are present in the cytosol and that phagosomal escape is dependent upon the ESX-1 secretion system [32,38]. This was confirmed by a FRET-based method dependent on β-lactamase production by Mtb in *ex vivo*-infected cells as well as in pulmonary phagocytic cells obtained from infected mice [33,34]. We report in the current study that the mycobacterial gene *Rv3167c* negatively regulates the escape of Mtb from the phagosome to the cytosol (Fig 4). Our study is the first to suggest that Mtb can exert temporal control on phagosomal escape as MtbΔ*Rv3167c* was found in the cytosol as early as 24h after infection while Mtb has been reported to access the cytosol much later in the infection process (4–5 days) [33]. We hypothesize that *Rv3167c* represses cytosolic escape at early stages when the bacterial load is low, favoring Mtb replication and establishment of infection. Genes involved in cytosolic escape could be induced once bacterial numbers reach about 20 per cell which seems to be an important threshold to switch on the cell escape program of Mtb [30]. Gene deletions resulting in increased cytosolic translocation from vacuolar compartments have been reported for other bacteria; for example, the *sdhA* (a Dot/Icm-secreted effector) mutant of *Legionella pneumophila* and the *sifA* (an SPI2-secreted effector) mutant of *Salmonella typhimurium* [81,82]. Deletion of the secreted phospholipase A abrogated the early escape of the *L*.*pneumophila sdhA* mutant [81]. The Mtb genome encodes four phospholipases (*plc A-D*), which could potentially contribute to the early escape of MtbΔ*Rv3167c*. The mechanisms involved in Mtb escape from the phagosome and eventual induction of host cell necrosis to exit the cell are difficult to study because of the slow kinetic of the process. Consequently, the early induction of cytosolic escape and necrosis by MtbΔ*Rv3167c* make it a useful model to study the host cell escape mechanisms of Mtb.

The escape of Mtb from the phagosome to the cytosol is closely followed by necrotic death of the host cells [32,33,83]. Consistently, we measured higher levels of cell death by necrosis in MtbΔ*Rv3167c*-infected cells (Figs 1A, 1B and S9E). Mtb may induce necrosis via the manipulation of host cell lipid mediators by favoring the production of the eicosanoid LXA_4_ [27]. In addition, the activation of the NLRP3-inflammasome was also shown to induce necrosis after Mtb infection [56]. We found that NLRP3 is dispensable for MtbΔ*Rv3167c*-mediated necrosis (S4C Fig). The regulation of cell death is complex and recently there have been major discoveries of signal transduction pathways for the regulation of programmed necrosis [1]. Using a combination of inhibitors and macrophages from knock-out mice, we screened for host factors required for MtbΔ*Rv3167c*-induced necrosis and ruled out the involvement of known programmed necrosis pathways (Figs 2, S4 and S5). Redundancy between the various necrosis-signaling modules may explain this result. For instance, both RIPK1-RIPK3 and caspase-1 activation are required for *S*.*tymphimurium*-induced necrotic cell death and blocking either one of the signaling pathways led only to a marginal inhibition of the death phenotype [43]. However, we found elevated mitochondrial ROS (mROS) to be required for the pro-necrotic phenotype of MtbΔ*Rv3167c* (Fig 6). Elevated mROS production could lead to increases in cytosolic ROS levels that may via lipid oxidation cause lysosomal permeabilization and cell death [1,84]. Lysosomal permeabilization has been implicated previously in necrosis induction by high bacillary loads of Mtb [61]. It is possible that MtbΔ*Rv3167c* may exploit a similar mechanism to kill host cells as increased lysosomal permeabilization was observed in cells infected with the mutant bacteria (Fig 2D). The Mm-mediated induction of necrosis after zebrafish infection also requires an increase in mROS [49]. Nevertheless, in contrast to our data (Figs 2A, S3B and S4A), Mm signals through the TNF/RIPK3 pathway to induce an increase of mROS and necrosis. The differences may reflect the variations in molecular pathogenesis pathways engaged by the human pathogen Mtb and the fish pathogen Mm.

Our data indicates involvement of diminished Akt activation in mitochondrial ROS generation in MtbΔ*Rv3167c*–infected cells (Fig 6E). The augmented phagosomal escape observed in MtbΔ*Rv3167c*-infected cells may allow for previously sequestered Mtb proteins to target mitochondria and trigger an increase in mROS generation. The mycobacterial type VII secretion system, ESX-5, is involved in the secretion of proteins containing Pro-Pro-Glu (PPE), Pro-Glu (PE) and polymorphic GC-rich sequences (PGRS) and has been implicated in cell lysis after Mtb escapes from the phagosome [39,85]. Interestingly, the ESX-5 substrates PE25 and PPE41 form a complex and induce necrosis [39,40]. Furthermore, the Mtb PE_PGRS33 protein, when ectopically expressed in a eukaryotic cell, localizes to the mitochondria and induces apoptotic and necrotic cell death [86]. The importance of these proteins in the context of infection with live bacteria has not been demonstrated yet. Another compelling target could be the secreted Mtb toxin CpnT which induces RIPK1-independent necrosis in Mtb-infected macrophages [87] via its NAD+ glycohydrolase activity [88].** ** It is possible that both mitochondrial localization of mycobacterial proteins and inhibition of Akt activation via Mtb proteins may both contribute to elevated mitochondrial ROS levels seen observed in MtbΔ*Rv3167c*-infected cells.

Autophagic clearance is a defense mechanism employed by host cells following detection of cytosolic pathogens. While macroautophagy (hereafter referred to as autophagy) is defined as the engulfment of cytosol by the autophagosome, selective autophagy describes the process in which autophagosome formation is directed towards a specific organelle, protein complex or microorganism by cargo receptor proteins (p62, NDP52, NBR1, Optineurin) [89–91]. Selective autophagy augments killing of intracellular mycobacteria, since reduced bacterial viability was seen following autophagy induction with IFNγ treatment of BCG-infected macrophages [92]. The relevance of selective autophagy for host defense against Mtb was demonstrated by the dramatically increased susceptibility of Atg5^-/-^ mice when compared to wild-type mice [66,93]. It was thus unexpected that MtbΔ*Rv3167c* was hypervirulent in the mouse model (Fig 7A) even though increased autophagy induction was observed in MtbΔ*Rv3167c*-infected cells compared to Mtb-infected controls (Fig 3C and 3D). We found that while MtbΔ*Rv3167c* induces autophagy, there was no increase in selective autophagy, as very few mycobacteria (both wild-type and mutant) co-localized with autophagosomes (Fig 3A and 3B). Previous studies have shown that Mtb has evolved mechanisms to avoid recruitment into the autophagosome [66,72,94–96]. Our results support this observation and show that this immune evasion strategy remains intact in MtbΔ*Rv3167c*. The increased autophagy seen in MtbΔ*Rv3167c*-infected cells is most likely a host stress response to an increased number of cytosolic bacteria [66]. Autophagy may dampen inflammation by negative regulation of the inflammasome and via the degradation of danger associated molecular patterns during host cell necrosis [97,98]. Atg5^-/-^ mice have higher basal level of inflammation when compared to wild-type mice [93] and Mtb-infected Atg5^-/-^ mice had increased levels of pulmonary pro-inflammatory cytokines and exhibited increased lung tissue damage [66,99]. It is thus possible that in the absence of autophagy induction, the increased inflammatory response seen in MtbΔ*Rv3167c*-infected mice (Fig 7E and 7F) would have been even stronger.

Unlike apoptosis, which benefits the host by reducing mycobacterial viability, Mtb-induced necrosis is beneficial to the pathogen allowing it to exit from infected cells and to disseminate [14,100]. Consistent with this concept was our finding that the necrosis-inducing MtbΔ*Rv3167c* strain was hypervirulent in mice and guinea pig. The hypervirulence of various clinical Mtb strains and Mtb deletion mutants has been reported previously [101]. For example, the Beijing strain HN878 was found to be more virulent than another member of the same family in immunocompetent mice [102]. Deletion of the *mce1* operon, two component response regulators *KdpDE*, *tcrXY* and the serine threonine protein kinases *pknH*, *pknE* and *pknI* rendered Mtb hypervirulent in mouse studies [103–107]. The presence of multiple anti-virulence genes in Mtb gives rise to the question: why would Mtb encode genes that suppress its virulence? While virulence may be defined as the ability of a pathogen to cause disease, an important aspect of virulence is successful transmission between hosts [108]. As Mtb has probably co-evolved with humans for more than 50,000 years, moderation of its virulence would have prevented elimination of the early existent small host populations thus maximizing transmission opportunities and improving persistence of the pathogen [109,110].

## Materials and Methods

### Materials

THP1 monocytes were obtained from ATCC (TIB 202). GFP tagged LC3 expressing THP1 monocytes (THP1 LC3GFP) were provided by Dr. John Kehrl (NIH). THP1shASC and THP1shcontrol cells were obtained from Dr. Jenny Ting (University of North Carolina). C57Bl6, *Nox2* ^*-/-*^, *Casp3* ^*-/-*^ and mCAT transgenic mice were obtained from Jackson Laboratories. *Ripk3* ^*-/-*^ mice were obtained from Genentech. *Casp1/11* ^*-/-*^ mice were provided by Dr. Denise Monack (Stanford School of Medicine). *Parp1* ^*-/-*^ mice were obtained from Dr. Ted Dawson (Johns Hopkins University). *Tnfr1* ^*-/-*^, *Il1r1* ^*-/-*^, *Irf3* ^*-/-*^ and *Ifnβ* ^*-/-*^ mice were provided by Dr. Alan Sher (NIH). Immortalized wildtype, *Nlrp3* ^*-/*^ and *Trif* ^*-/-*^ *MyD88* ^*-/-*^ BMDMs were provided by Dr. Igor Brodsky (University of Pennsylvania). Atg5^fl/fl^ LysM Cre^+^ (Atg5^-/-^) and Atg5^fl/fl^ LysM Cre^-^ (Atg5^+/+^) mice were obtained from Dr. Herbert Virgin IV (Washington University School of Medicine). zVAD FMK, Necrostatin 1, MAPK inhibitors (SP600125, SB203580), Akt activator (sc-79) and DPI were purchased from Calbiochem. BafilomycinA1, glutathione and N-acetyl cysteine were sourced from Sigma. 3-MA was purchased from Tocris Biosciences.

### Generation of mutant and complement strains

*Rv3167c* was deleted in *M*. *tuberculosis* H37Rv using a specialized phage transduction strategy described previously [111]. Gene deletion was confirmed by RT-PCR as well as by southern blotting. The probes used were labeled with biotin using BrightStar Psoralen-Biotin Kit. Genomic DNA was digested with EcoRI. The DNA fragments were separated by agarose gel electrophoresis, transferred to charged nylon membrane, and denatured with 0.4N NaOH. The probe was denatured at 90°C for 10 min in the presence of 10mM EDTA and hybridized to the membrane at 55°C for 16h in hybridization buffer (AlkPhos Direct hybridization buffer with 0.5M NaCl). The membrane was washed and the probe was detected using a BrightStar BioDetect Nonisotopic Detection Kit. For generating the complement strain, *Rv3167c* gene sequence including 60bp upstream was cloned into the episomal plasmid pMV261, electroporated into the *Rv3167c* mutant strain and plated on 7H10 plates with 40μg/ml kanamycin.

### Bacterial culture

Bacterial strains were grown in 7H9 medium supplemented with 10% ADC, 0.5% glycerol and 0.05% Tween 80. Hygromycin (50μg/ml) and kanamycin (40μg/ml) were added to the mutant and complement cultures respectively. For infection, cultures with an OD_600_ between 0.6–0.8 (corresponding to the late log phase of growth) were pelleted and resuspended in 0.05% PBS-Tween 80 prior to addition to cells.

### Determination of *in vitro* and *ex vivo* bacterial growth rate

To measure *in vitro* bacterial growth, bacteria were added to 7H9 medium to obtain a starting OD_600_ of 0.01. OD_600_ measurements were made at 24h intervals until 7 days. *Ex vivo* bacterial growth was determined by infecting THP1 macrophages and lysing them at the indicated timepoints with 0.1% Triton X 100. Appropriate dilutions were plated on 7H11 medium in triplicate. Inoculated plates were incubated at 37°C and colonies were counted approximately 2 weeks after plating.

### Cell culture and infection

THP1 monocytes were maintained in RPMI 1640 supplemented with 10% heat inactivated FCS. Cells were differentiated with 20ng/ml PMA for 20–24 hours, washed and infected in growth medium containing 5% human serum. Bacteria were added to cells at MOI 3 for 4 hours at 37°C, extracellular bacteria were removed by PBS washes and chase medium containing 100μg/ml gentamicin was added. BMDMs were prepared from cells obtained from femurs and tibia of various mouse strains and cultured in DMEM supplemented with 10% heat inactivated FCS, 25% L929 supernatant and 1% Penicillin-Streptomycin. Growth medium was replaced with DMEM containing 10% non-heat inactivated FCS for 4h and cells were infected at MOI 10 in same medium in the manner described above. Chase media contained 10% L929 supernatant in order to avoid cell death induction due to cytokine withdrawal. Immortalized BMDMs were maintained in DMEM containing 10% heat inactivated FCS and infected in media similar to that used for primary BMDMs. Human monocyte derived macrophages (hMDMs) were prepared from elutriated monocyte fractions obtained from NIH blood bank. Monocyte fractions were seeded in serum free RPMI for one hour. Non-adherent cells were removed and adherent cells were differentiated in RPMI medium containing 5% off-the-clot AB human serum (Gemini) and 10ng/ml human MCSF (Peprotech) for 7 days. Inhibitors (with the exception of 3-MA) were added to cells one hour prior to infection and included in chase medium. 3-MA was added only to chase medium. For all experiments, 0h time point refers to end of infection period when cells have been exposed to bacteria for 4 hours.

### Cell death assays

Cells were stained with 1μg/ml propidium iodide (PI) (Sigma-Aldrich) for 10 minutes at room temperature and analyzed by flowcytometry. For TUNEL stain, cells were fixed in 4% paraformaldehyde overnight, stained as per manufacturer’s instructions (Roche) and examined by either flow cytometry or fluorescence microscopy. Hypodiploid stain was performed using PI/RNase staining buffer (BD Pharmingen) following overnight fixation in 70% ethanol as per manufacturer’s instructions. For all flow cytometry analyses, at least 10,000 cells were acquired (BD Accuri C6). Toxilight assay to measure adenylate kinase release from cells was performed as per manufacturers instructions.

### Autophagy analysis

Autophagy induction in THP1 LC3GFP expressing cells was analyzed as described previously [67]. Briefly, cells were permeabilized with 0.05% saponin for 5 minutes, washed and resuspended in PBS containing 5% FCS. Permeabilization resulted in loss of cytosolic LC3I while LC3II bound to autophagosome membranes were retained, which was measured by flow cytometry (50,000 cells acquired, BD Accuri C6). For immunofluorescence analysis, bacteria were stained with 0.4mg/ml AF647-NHS ester (Molecular Probes) in 0.1M sodium bicarbonate solution for 30 minutes at 37°C and used for infecting cells on slides. At specified time points, cells were fixed with 4% paraformaldehyde overnight, stained with Hoechst 33342 and analyzed by confocal microscopy (Zeiss LSM710).

### Immunoblotting

Cell lysates were obtained by lysing cells with RIPA buffer containing protease (Complete, Mini EDTA free, Roche) and phosphatase inhibitor cocktails (PhosStop, Roche) followed by centrifugation at 12,000g for 5 minutes. Pierce BCA protein assay kit (Thermo Scientific) was used to measure protein concentrations to ensure equivalent loading. Antibodies against phosphorylated and total Akt and MAPKs, PARP, tubulin and GFP were purchased from Cell Signaling and used at 1:1000 dilution. Anti LC3 antibody was purchased from Epitomics and used at 1:2500 dilution. Densitometric analysis was performed using ImageJ software.

### CCF4 FRET assay

To detect mycobacterial escape from the phagosome, the CCF4 FRET assay was performed as described previously [34]. Briefly, cells were stained with 8μM of CCF4 (Invitrogen) in EM buffer (120mM NaCl, 7mM KCl, 1.8mM CaCl_2_, 0.8mM MgCl_2_ 5mM glucose, 25mM Hepes, pH7.3) containing 2.5μM of probenecid (Sigma-Aldrich) for 1.5 hours at room temperature. Live populations were distinguished from dead ones by addition of Live/Dead Fixable Red stain (Invitrogen) for 30 minutes at room temperature. After staining cells were fixed with 4% PFA overnight and analyzed by flow cytometry (BD FACS CantoII). 40,000 cells were acquired and post acquisition analysis done using FlowJo software (Treestar, OR). For estimation of bacterial β-lactamase activity, bacteria were resuspended in PBS containing 50μg/ml porcine esterase liver extract and 100nM CCF4-AM and incubated at 37°C for 12h. Fluoresence measurements were made using Biotek Synergy 4 microplate reader.

### Measurement of ROS generation

For measurement of ROS levels in BMDMs, cells were infected as described previously [78]. At indicated time points after infection, cells were harvested and stained with 10μM CM-H2DCFDA (Molecular Probes) or 1.25μM MitoSOX Red (Molecular Probes) for 30 minutes at 37°C in HBSS. Cells were analyzed by flow cytometry (at least 10,000 cells acquired, BD Accuri C6) after HBSS wash.

### DIOC_6_ staining

Cells were stained with 40 nmol of DIOC_6_ stain (Molecular Probes) at 37°C for 15 minutes, washed and analyzed by flow cytometry (10,000 cells acquired, BD Accuri C6).

### Electron microscopy

THP1 cells were fixed in 2% gluteraldehyde and 2% paraformaldehyde in 0.1M sodium cacodylate buffer pH7.4 for 1 hour. They were carefully pelleted and re-suspended in 2% paraformaldehyde for several hours, followed by rinsing in 0.1M sodium cacodylate buffer and pelleted in 2% agar in the same buffer. The samples were post fixed in 1% osmium tetroxide in 0.1M sodium cacodylate buffer, rinsed in distilled water and, en bloc stained in 2% aqueous uranyl acetate for a further hour. They were then rinsed and dehydrated in an ethanol series (50% to 100%) followed by resin infiltration Embed 812 (Electron Microscopy Sciences) and baked overnight at 60°C. Hardened blocks were cut using a Leica UltraCut UC7. 60nm sections were collected on formvar/carbon coated nickel grids and contrast stained using 2% uranyl acetate and lead citrate. Grids were all viewed in a FEI Tencai Biotwin TEM at 80Kv. Images were taken using Morada CCD and iTEM (Olympus) software.

Embedding and sectioning was performed at the electron microscopy core facility at the Yale School of Medicine.

### Animal studies

C57Bl/6 mice were infected with 100 CFU of each of the various bacterial strains grown to late log phase via the aerosol route using a Glas-Col full body inhalation exposure system. At the indicated time points, 3 mice per group were sacrificed and bacterial load was determined by homogenizing the organs in PBS and plating serial dilutions on 7H11 plates. Lung homogenate supernatants were used for cytokine analysis using the Luminex MAGPIX platform (R&D Bioscience). Superior lobes of the lungs were fixed in 10% buffered formalin for histopathology. Paraffin embedding, sectioning and hematoxylin and eosin (H and E) staining were performed by AML Labs, Baltimore. Total lung area and areas of inflamed regions in H and E stained lung sections were quantified using ImageJ.

Female outbred Hartley guinea pigs (250-300g) were purchased from Charles River Labs (Wilmington, MA). Animals were infected with each of the three Mtb strains via aerosol using a Madison chamber aerosol generation device (University of Wisconsin, Madison, WI) calibrated to deliver ~3 log_10_ CFU in the lungs. Four animals from each group were sacrificed on day 1 and day 28 post-infection. The lungs were homogenized, as previously described, and the lung homogenates were plated on 7H11 Middlebrook agar and incubated at 37°C for 4 weeks before final CFU counts were determined [112].

Alveolar macrophages were harvested by bronchoalveolar lavage (BAL) as described previously [113]. Briefly, cold PBS with 3% FCS was instilled into the lungs following insertion into the trachea of an 18-gauge cannula fixed to a 20-ml syringe. The cells were pelleted by centrifugation at 380g for 10 min, washed twice with RPMI-1640 supplemented with 10% FCS, and 10μM 2-mercaptoethanol (RPMI complete medium), and resuspended in 1 ml RPMI complete medium. Following transport from Johns Hopkins to University of Maryland on ice, viable cells were enumerated by the trypan blue exclusion method and seeded in RPMI complete medium overnight. Adherent cells were infected in RPMI medium containing 5% FCS at specified MOI for 4 hours at 37°C, extracellular bacteria were removed by PBS washes and chase medium containing 100μg/ml gentamicin was added.

### Statistical analysis

Statistical analysis was performed using GraphPad Prism version 6.0 software. Data is presented as mean ± S.E.M. of three independent experiments and one-way ANOVA with Tukey post-test was used unless mentioned otherwise in the figure legends. p-value significance is as follows—*- ≤0.05, ** - ≤0.01, *** - ≤0.001, **** - 0.0001.

### Ethics statement

All animals were handled in accordance with the NIH guidelines for housing and care of laboratory animals and the studies were approved by the Institutional Animal Care and Use Committees at the University of Maryland, College Park (protocol no—R-12-55) and Johns Hopkins University School of Medicine (protocol no—GP12M88).

## Supporting Information

S1 Fig Identification of *Rv3167c* as an anti cell death gene. (A) Insert of cosmid J21. Position of deletion mutants that induce higher cell death compared to Mtb indicated. The 7/10 region contains *Rv3167c*. The image has been adapted from the Tuberculist website (B) Cell death induction by Mtb deletion mutants in the J21 cosmid determined by TUNEL staining and flow cytometry (mean ± S.E.M, n = 6) (C) Screening of individual gene deletion mutants in the 7/10 region of the J21 cosmid for increased cell death induction compared to Mtb performed by TUNEL staining and flow cytometry (mean ± S.E.M, n = 3).
(TIF)

S2 Fig Generation of *Rv3167c* mutant (MtbΔ*Rv3167c*) in Mtb. (A) A hygromycin cassette was introduced into the Mtb H37Rv genome by specialized transduction to generate MtbΔ*Rv3167c*. An episomal plasmid with *Rv3167c* expression under the control of a constitutively active promoter was used to generate the complement strain MtbΔ*Rv3167c*-C. The sizes of fragments obtained after digestion of genomic DNA with EcoRI and using a specific probe for southern blotting are indicated. (B) Knockout and complementation of *Rv3167c* was confirmed by southern blotting. (C) Deletion of *Rv3167c* confirmed by RT-PCR. A non-related mutant (Δ3165c) was used as a control to demonstrate primer specificity. (D) Replication of bacterial strains *ex vivo* was determined by lysing infected THP1 cells and plating lysates on 7H11 medium at indicated times (mean ± S.E.M, n = 9). (E) *In vitro* growth rate of bacterial strains in 7H9 medium was measured every 24h (mean ± S.E.M, n = 9).
(TIFF)

S3 Fig Efficacy of zVAD fmk and Necrostatin-1. (A) Efficacy of the pan caspase inhibitor zVAD-fmk (40μM) was assessed by inhibition of camptothecin-mediated apoptosis in THP1 cells measured by TUNEL staining and flow cytometry (mean ± S.E.M, n = 3). (B) BMDMs were treated with the RIPK1 inhibitor necrostatin-1 (Nec1) (100μM) one hour prior to and throughout the infection and necrosis induction measured by PI staining at 24h (mean ± S.E.M, n = 3). (C) Necrostatin-1 (Nec1, 100μM) efficacy was determined by inhibition of LPS and zVAD-fmk induced cell death (RIPK1 dependent) in BMDMs measured by PI staining and flowcytometry (mean ± S.E.M, n = 3). (D) *RipK3* ^*-/-*^ BMDMs were treated with the pan caspase inhibitor zVAD FMK one hour prior to and throughout the infection and necrosis induction measured by PI staining at 24h (mean ± S.E.M, n = 3).
(TIFF)

S4 Fig MtbΔ*Rv3167c* mediated necrosis is independent of TNF, IL1, NLRP3 inflammasome and type I IFN. (A) Necrosis induction in WT and *Tnfr1* ^*-/-*^ BMDMs was determined by PI staining and flow cytometry at 72h (mean ± S.E.M, n = 3). (B) Necrosis induction in WT and *IL1r1* ^*-/-*^ BMDMs was determined by PI staining and flow cytometry at 72h (mean ± S.E.M, n = 6). (C) Necrosis induction in immortalized WT and *Nlrp3* ^*-/-*^ BMDMs was determined by PI staining and flow cytometry at 24h (mean ± S.E.M, n = 3). (D) Necrosis induction in THP1 shASC and control cells was determined by Toxilight assay at 48h (mean ± S.E.M, n = 9) (E) Necrosis induction in WT and *Irf3* ^*-/-*^ BMDMs was determined by PI staining and flow cytometry at 48h (mean ± S.E.M, n = 3) (F) Necrosis induction in WT and *Ifnβ* ^*-/-*^ BMDMs was determined by PI staining and flow cytometry at 48h (mean ± S.E.M, n = 8).
(TIFF)

S5 Fig Mtb*ΔRv3167c* mediated necrosis is independent of TLR signaling. Necrosis induction in (A) WT and *Trif* ^*-/-*^, (B) WT and *MyD88* ^*-/-*^ and (C) immortalized WT and *Trif* ^*-/-*^ *MyD88* ^*-/-*^ BMDM’s was determined by PI staining and flow cytometry at 48h (mean ± S.E.M, n = 3).
(TIFF)

S6 Fig MtbΔ*Rv3167c* does not inhibit autophagosome maturation. (A) Accumulation of LC3II GFP in MtbΔ*Rv3167c* infected THP1 LC3GFP cells treated with Bafilomycin (BafA1, 250nM) examined by flow cytometry at 16h (mean ± S.E.M, n = 6). (B) Free GFP generated during lysosomal degradation of LC3II GFP detected by western blotting in whole cell lysates. Image is representative of three independent experiments. (C) Necrosis induction in presence of autophagy inhibitor 3-MA was determined by Toxilight assay at 24h (mean ± S.E.M, n = 4).
(TIF)

S7 Fig Mtb, MtbΔ*Rv3167c* and MtbΔ*Rv3167c*-C have similar β-lactamase activity *in vitro*. β-lactamase activity of the indicated bacterial strains was determined by incubating bacterial cultures with CCF4-AM and measuring fluorescence emission at 450nm and 535nm. Data is represented as fold change over CCF4-AM incubated in absence of bacteria. ΔblaC is a *blaC* deleted H37Rv strain [114].
(TIFF)

S8 Fig Necrosis and autophagy induction by MtbΔ*Rv3167c* is dependent on ROS. (A) Effect of the flavoprotein inhibitor DPI (10μM) on necrosis induction in THP1 cells was determined by the Toxilight assay at 24h (mean ± S.E.M, n = 6). (B) Effect of the antioxidants glutathione (15mM) and N-acetyl cysteine (NAC, 10mM) on necrosis induction in THP1 cells was determined by TUNEL staining and fluorescence microscopy at 24h (mean ± S.E.M, n = 9). (C) Loss of mitochondrial membrane potential was determined by DIOC_6_ staining at the indicated time points (mean ± S.E.M, n = 9). (D) Effect of DPI (10μM) on autophagy induction in THP1 LC3GFP cells was determined by flow cytometry (mean ± S.E.M, n = 6).
(TIFF)

S9 Fig MtbΔ*Rv3167c* is hypervirulent in SCID mice and guinea pigs. (A) Survival of SCID mice infected via the aerosol route with 100 CFU of bacteria (n = 5). (B) Bacterial uptake by SCID mice was determined by plating lung homogenates prepared at day 1 (mean ± S.E.M, n = 3). (C) Guinea pigs were infected via aerosol route. Bacterial uptake by guinea pigs was determined by plating lung homogenates prepared at 1d (means ± S.E.M, n = 4). (D) Lung burden in guinea pigs at 28 days (mean ± S.E.M, n = 4). (E) Cell death induction by MtbΔ*Rv3167c* in guinea pig alveolar macrophages infected *ex vivo* was determined by TUNEL staining and microscopy (mean ± S.E.M, n = 3).
(TIF)
