# Supplementary figures and images for: Identification of a Transcription Factor That Regulates Host Cell Exit and Virulence of Mycobacterium tuberculosis
Source: PLoS Pathog. 2016 May 18;12(5):e1005652. doi: 10.1371/journal.ppat.1005652 (PMC4871555; doi:10.1371/journal.ppat.1005652)

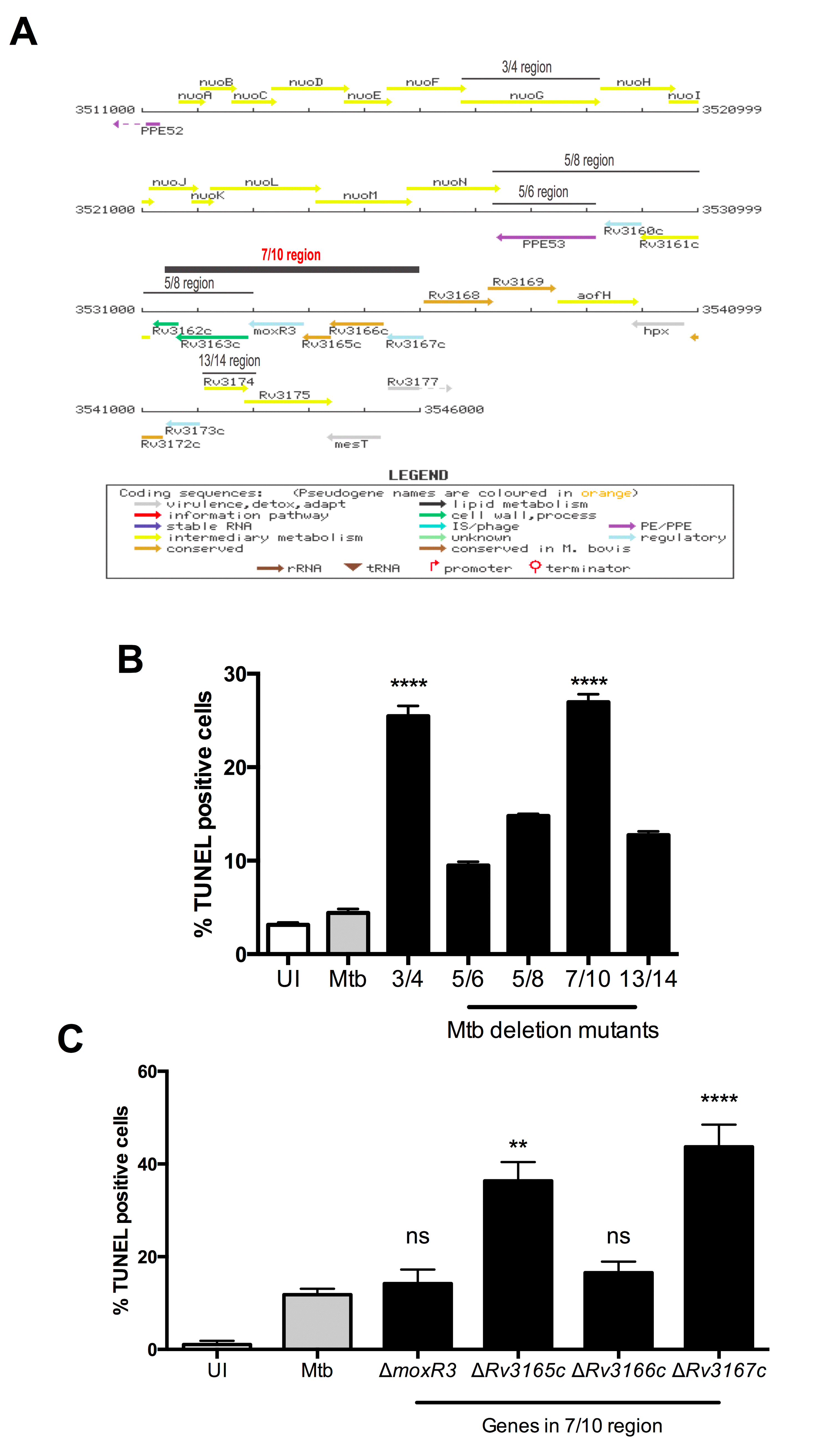

Supplement: S1 Fig — (A) Insert of cosmid J21. Position of deletion mutants that induce higher cell death compared to Mtb indicated. The 7/10 region contains Rv3167c. The image has been adapted from the Tuberculist website (B) Cell death induction by Mtb deletion mutants in the J21 cosmid determined by TUNEL staining and flow cytometry (mean ± S.E.M, n = 6) (C) Screening of individual gene deletion mutants in the 7/10 region of the J21 cosmid for increased cell death induction compared to Mtb performed by TUNEL staining and flow cytometry (mean ± S.E.M, n = 3). (TIF) [file ppat.1005652.s001.tif]

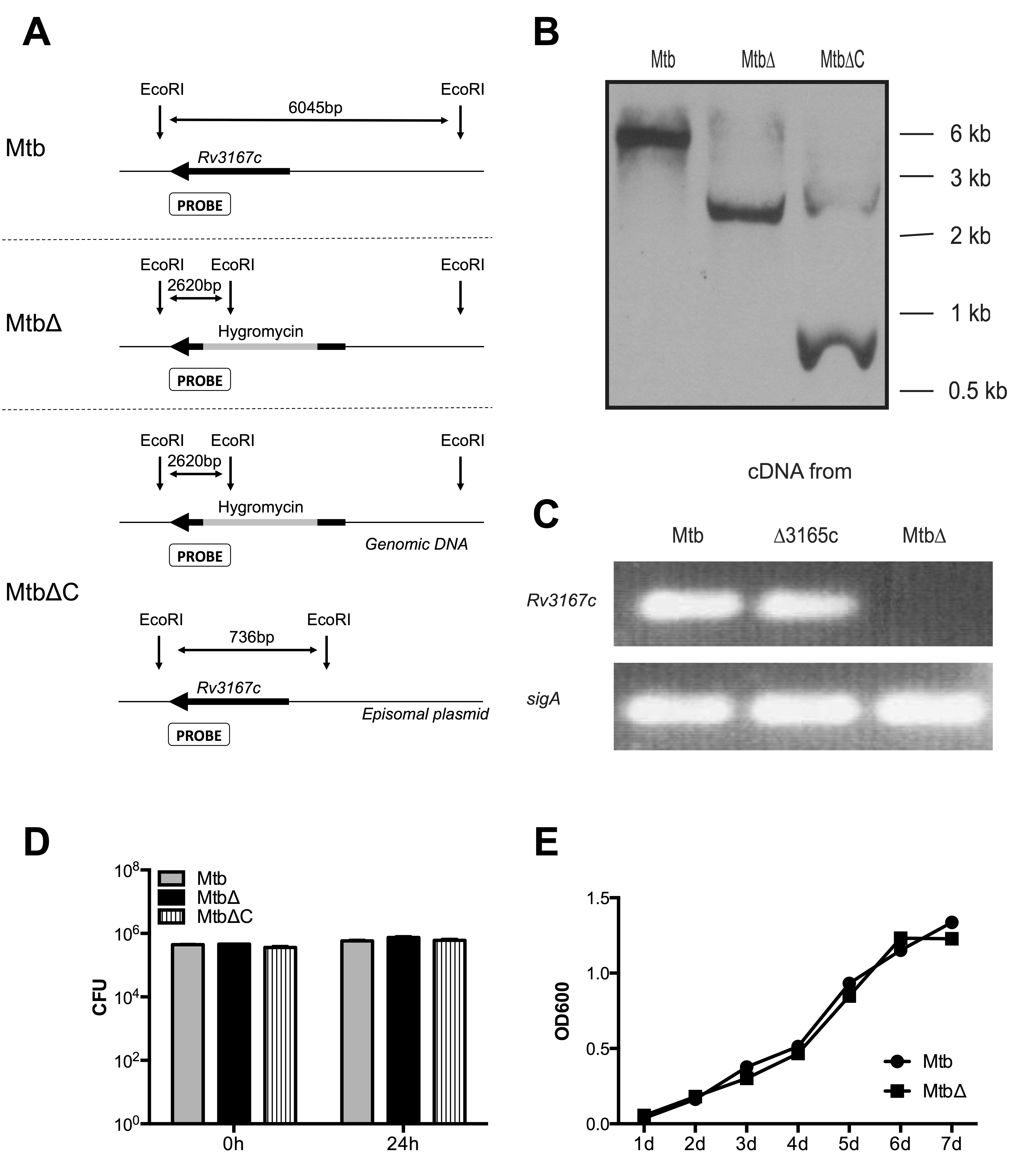

Supplement: S2 Fig — (A) A hygromycin cassette was introduced into the Mtb H37Rv genome by specialized transduction to generate MtbΔRv3167c. An episomal plasmid with Rv3167c expression under the control of a constitutively active promoter was used to generate the complement strain MtbΔRv3167c-C. The sizes of fragments obtained after digestion of genomic DNA with EcoRI and using a specific probe for southern blotting are indicated. (B) Knockout and complementation of Rv3167c was confirmed by southern blotting. (C) Deletion of Rv3167c confirmed by RT-PCR. A non-related mutant (Δ3165c) was used as a control to demonstrate primer specificity. (D) Replication of bacterial strains ex vivo was determined by lysing infected THP1 cells and plating lysates on 7H11 medium at indicated times (mean ± S.E.M, n = 9). (E) In vitro growth rate of bacterial strains in 7H9 medium was measured every 24h (mean ± S.E.M, n = 9). (TIFF) [file ppat.1005652.s002.tiff]

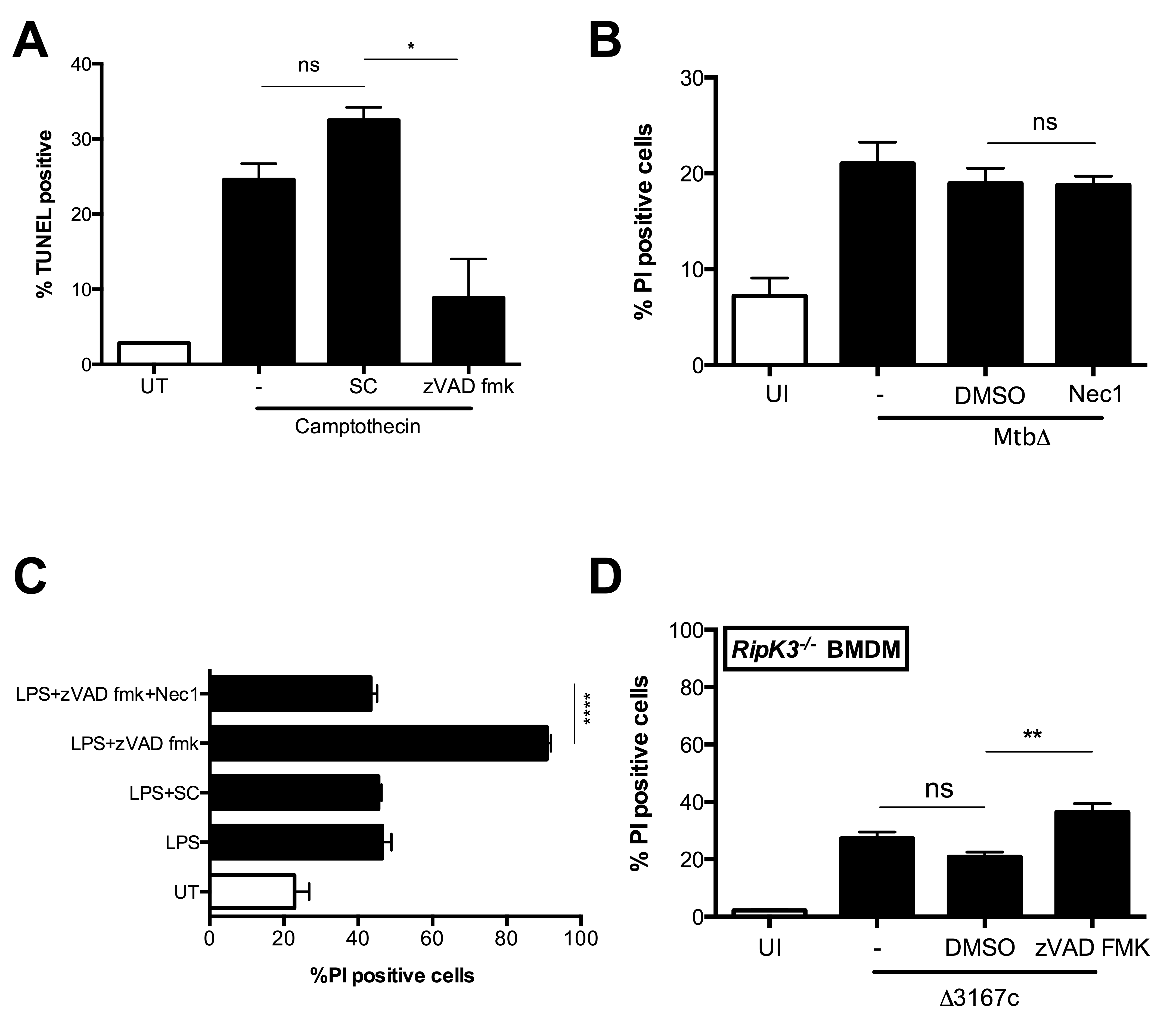

Supplement: S3 Fig — (A) Efficacy of the pan caspase inhibitor zVAD-fmk (40μM) was assessed by inhibition of camptothecin-mediated apoptosis in THP1 cells measured by TUNEL staining and flow cytometry (mean ± S.E.M, n = 3). (B) BMDMs were treated with the RIPK1 inhibitor necrostatin-1 (Nec1) (100μM) one hour prior to and throughout the infection and necrosis induction measured by PI staining at 24h (mean ± S.E.M, n = 3). (C) Necrostatin-1 (Nec1, 100μM) efficacy was determined by inhibition of LPS and zVAD-fmk induced cell death (RIPK1 dependent) in BMDMs measured by PI staining and flowcytometry (mean ± S.E.M, n = 3). (D) RipK3 -/- BMDMs were treated with the pan caspase inhibitor zVAD FMK one hour prior to and throughout the infection and necrosis induction measured by PI staining at 24h (mean ± S.E.M, n = 3). (TIFF) [file ppat.1005652.s003.tiff]

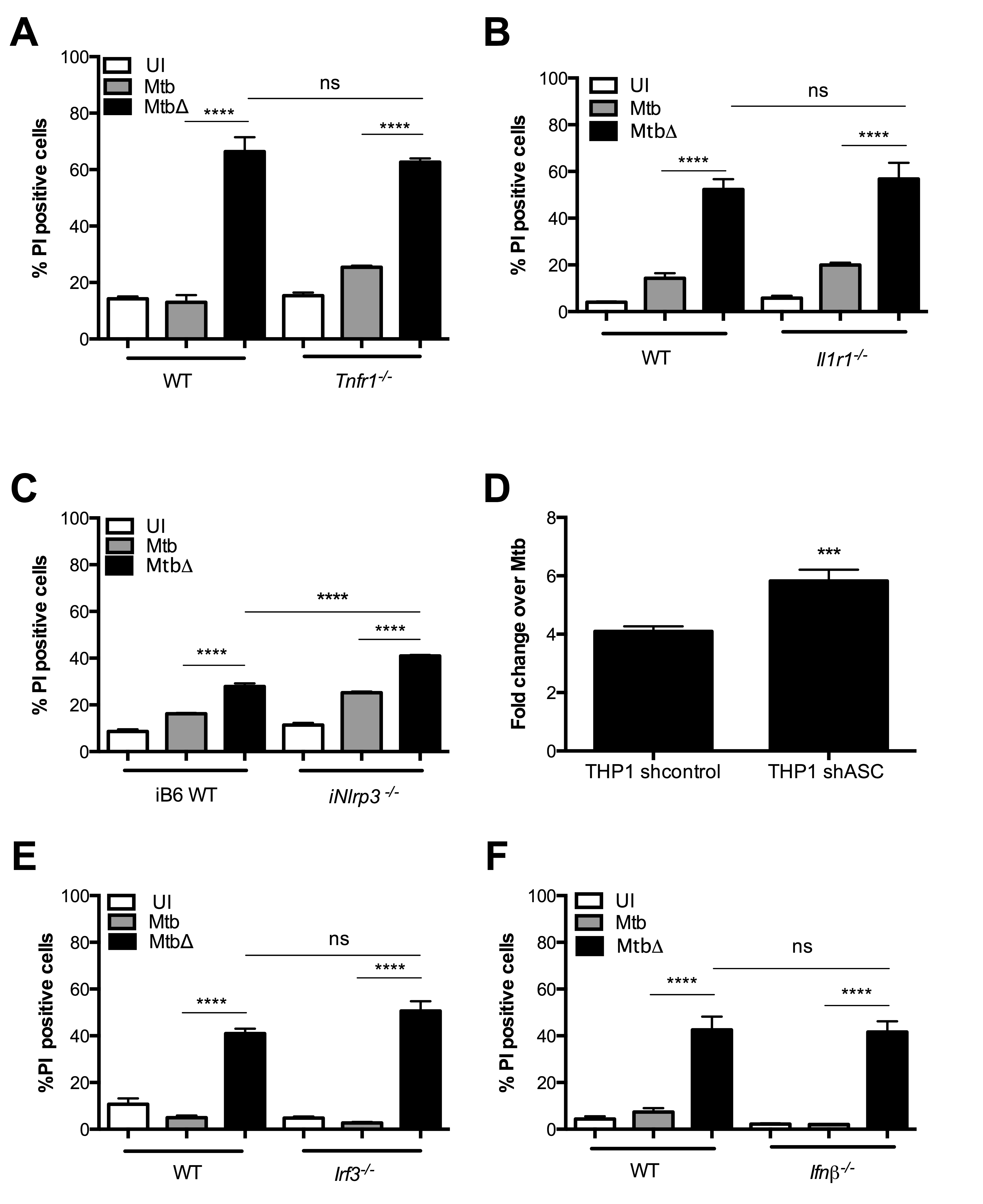

Supplement: S4 Fig — (A) Necrosis induction in WT and Tnfr1 -/- BMDMs was determined by PI staining and flow cytometry at 72h (mean ± S.E.M, n = 3). (B) Necrosis induction in WT and IL1r1 -/- BMDMs was determined by PI staining and flow cytometry at 72h (mean ± S.E.M, n = 6). (C) Necrosis induction in immortalized WT and Nlrp3 -/- BMDMs was determined by PI staining and flow cytometry at 24h (mean ± S.E.M, n = 3). (D) Necrosis induction in THP1 shASC and control cells was determined by Toxilight assay at 48h (mean ± S.E.M, n = 9) (E) Necrosis induction in WT and Irf3 -/- BMDMs was determined by PI staining and flow cytometry at 48h (mean ± S.E.M, n = 3) (F) Necrosis induction in WT and Ifnβ -/- BMDMs was determined by PI staining and flow cytometry at 48h (mean ± S.E.M, n = 8). (TIFF) [file ppat.1005652.s004.tiff]

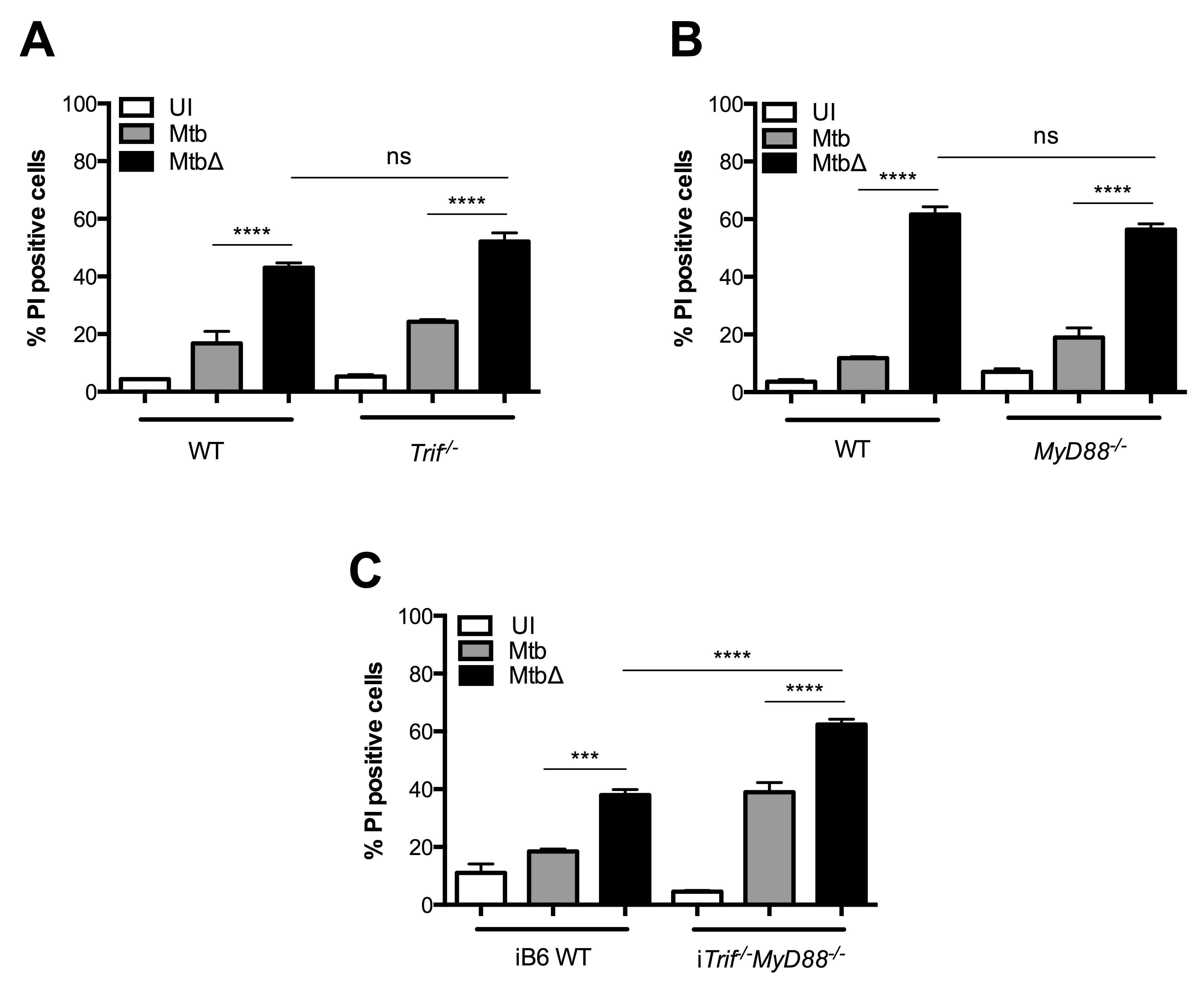

Supplement: S5 Fig — Necrosis induction in (A) WT and Trif -/-, (B) WT and MyD88 -/- and (C) immortalized WT and Trif -/- MyD88 -/- BMDM’s was determined by PI staining and flow cytometry at 48h (mean ± S.E.M, n = 3). (TIFF) [file ppat.1005652.s005.tiff]

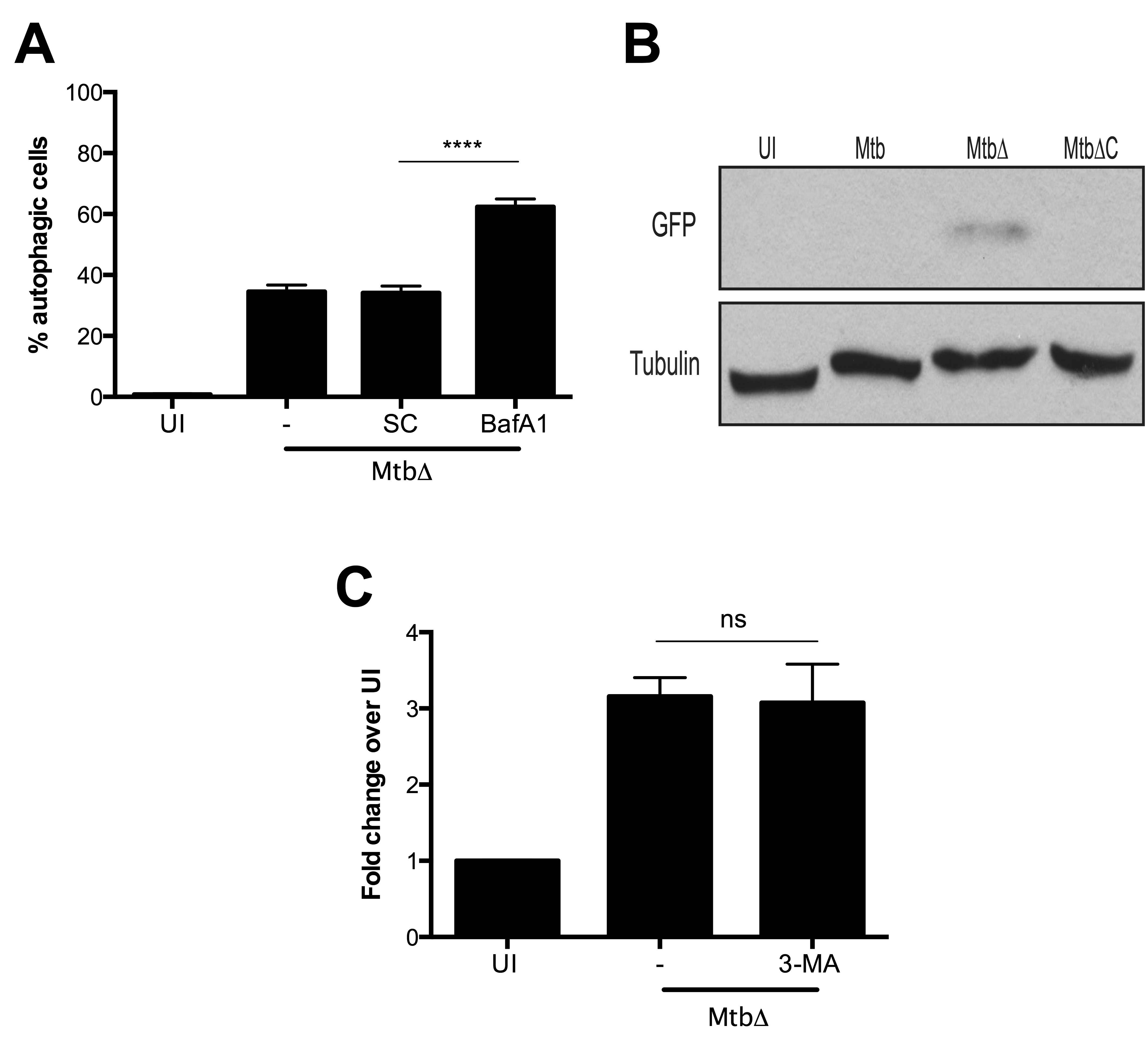

Supplement: S6 Fig — (A) Accumulation of LC3II GFP in MtbΔRv3167c infected THP1 LC3GFP cells treated with Bafilomycin (BafA1, 250nM) examined by flow cytometry at 16h (mean ± S.E.M, n = 6). (B) Free GFP generated during lysosomal degradation of LC3II GFP detected by western blotting in whole cell lysates. Image is representative of three independent experiments. (C) Necrosis induction in presence of autophagy inhibitor 3-MA was determined by Toxilight assay at 24h (mean ± S.E.M, n = 4). (TIF) [file ppat.1005652.s006.tif]

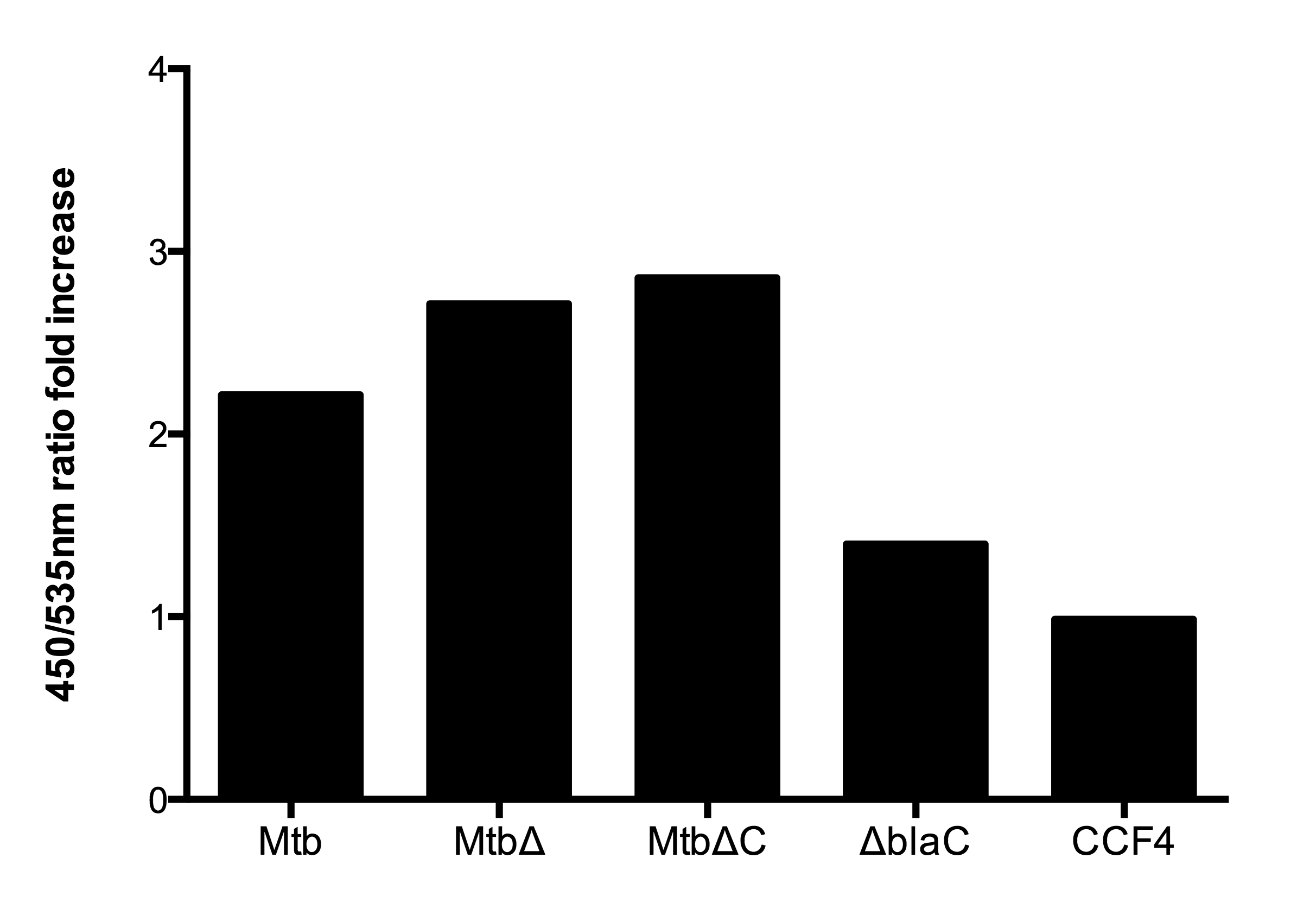

Supplement: S7 Fig — β-lactamase activity of the indicated bacterial strains was determined by incubating bacterial cultures with CCF4-AM and measuring fluorescence emission at 450nm and 535nm. Data is represented as fold change over CCF4-AM incubated in absence of bacteria. ΔblaC is a blaC deleted H37Rv strain [114]. (TIFF) [file ppat.1005652.s007.tiff]

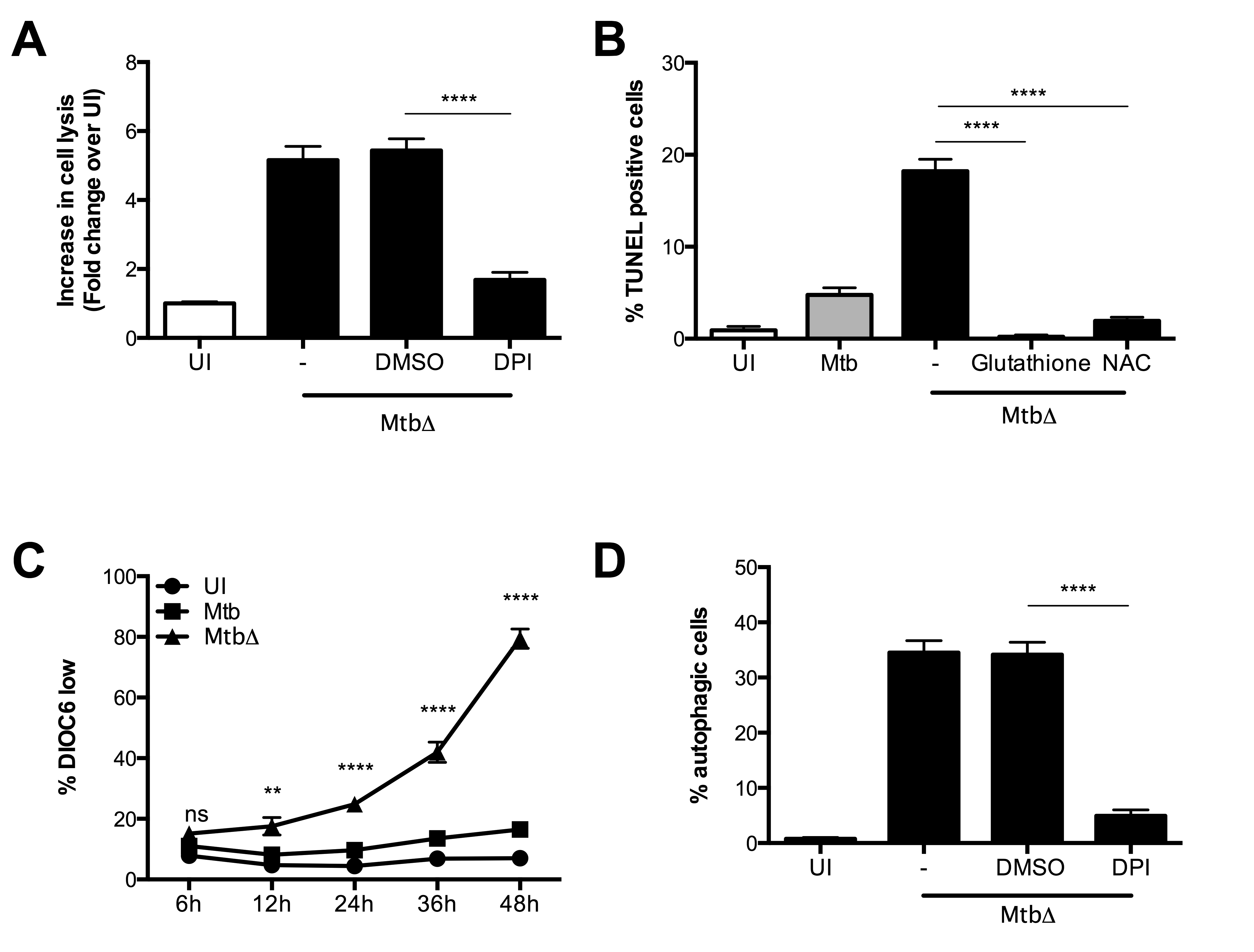

Supplement: S8 Fig — (A) Effect of the flavoprotein inhibitor DPI (10μM) on necrosis induction in THP1 cells was determined by the Toxilight assay at 24h (mean ± S.E.M, n = 6). (B) Effect of the antioxidants glutathione (15mM) and N-acetyl cysteine (NAC, 10mM) on necrosis induction in THP1 cells was determined by TUNEL staining and fluorescence microscopy at 24h (mean ± S.E.M, n = 9). (C) Loss of mitochondrial membrane potential was determined by DIOC6 staining at the indicated time points (mean ± S.E.M, n = 9). (D) Effect of DPI (10μM) on autophagy induction in THP1 LC3GFP cells was determined by flow cytometry (mean ± S.E.M, n = 6). (TIFF) [file ppat.1005652.s008.tiff]

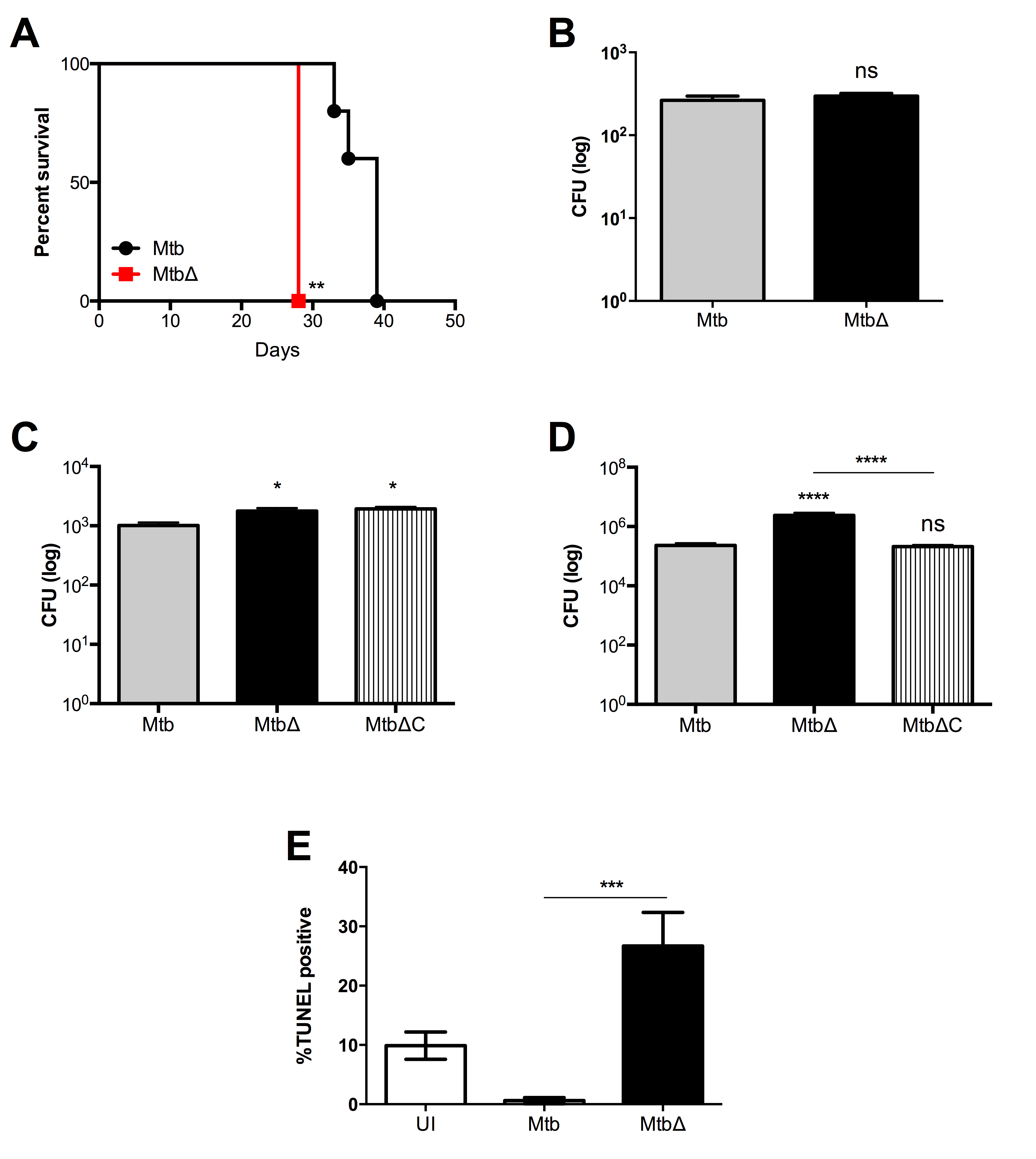

Supplement: S9 Fig — (A) Survival of SCID mice infected via the aerosol route with 100 CFU of bacteria (n = 5). (B) Bacterial uptake by SCID mice was determined by plating lung homogenates prepared at day 1 (mean ± S.E.M, n = 3). (C) Guinea pigs were infected via aerosol route. Bacterial uptake by guinea pigs was determined by plating lung homogenates prepared at 1d (means ± S.E.M, n = 4). (D) Lung burden in guinea pigs at 28 days (mean ± S.E.M, n = 4). (E) Cell death induction by MtbΔRv3167c in guinea pig alveolar macrophages infected ex vivo was determined by TUNEL staining and microscopy (mean ± S.E.M, n = 3). (TIF) [file ppat.1005652.s009.tif]
